# Comprehensive index analysis approach for ecological and human health risk assessment of a tributary river in Bangladesh

**DOI:** 10.1016/j.heliyon.2024.e32542

**Published:** 2024-06-06

**Authors:** Priyanka Dey Suchi, Md Aftab Ali Shaikh, Badhan Saha, Mohammad Moniruzzaman, Md Kamal Hossain, Afroza Parvin, Afsana Parvin

**Affiliations:** a Bangladesh Council of Scientific and Industrial Research (BCSIR), Dhaka-1205, Bangladesh; b Department of Chemistry, University of Dhaka, Dhaka-1000, Bangladesh

**Keywords:** Heavy metal pollution index, Contamination index, Nemerow pollution index, Human health hazard index, Ecological risk index, Water quality index, Turag River

## Abstract

This study examined the water quality of the Turag River, an important tributary river in Dhaka, Bangladesh in terms of physicochemical characteristics and heavy metal contamination to assess the potential risks to both ecological systems and human health. The majority of the water samples complied with the acceptable limits established by the World Health Organization (WHO) for various parameters including pH, electrical conductivity (EC), total dissolved solids (TDS), dissolved oxygen (DO), chemical oxygen demand (COD), sodium adsorption ratio (SAR), and magnesium adsorption ratio (MAR), except total hardness (TH). The sodium (Na), potassium (K), calcium (Ca), magnesium (Mg), chloride (Cl^−^)^,^ fluoride (F^−^), nitrate (NO_3_^−^), and sulfate (SO_4_^2−^) levels in the water samples were found to be within acceptable ranges for most cases. Moreover, heavy metals including lead (Pb), cadmium (Cd), chromium (Cr), nickel (Ni), iron (Fe), manganese (Mn), zinc (Zn), copper (Cu), arsenic (As), selenium (Se), and mercury (Hg) were analyzed and their mean concentrations (μg/L) were found in the order of Fe (244.72 ± 214.35) > Mn (28.93 ± 29.64) > Zn (22.97 ± 10.93) > Cu (8.28 ± 5.99) > Hg (8.23 ± 6.58) > As (1.34 ± 0.39) > Ni (1.20 ± 0.38) > Cr (0.67 ± 0.85) > Pb (0.61 ± 0.72) > Se (0.42 ± 0.48) > Cd (0.13 ± 0.09) which were within the acceptable limit, except Hg. The cumulative effect of all heavy metals was assessed through the heavy metal pollution index (HPI), contamination degree (C_d_), and nemerow pollution index (P_N_). The mean value of HPI (682.38 ± 525.68) crossed the critical index value of 100, indicating an elevated level of pollution. The mean value of C_d_ (8.763 ± 6.48) indicates a low-moderate-significant level of contamination due to an elevated level of Hg, and for the P_N_ it was found 174.27 ± 146.66, indicating a high level of pollution due to high level of Fe. Ecological risk index (ERI) indicated low levels of risk for Pb, Cd, Cr, Ni, Fe, Mn, As, Se, Cu, and Zn but a significantly high risk for Hg. The water was classified as good to excellent based on its physicochemical properties (pH, EC, TDS, COD, DO, F^−^, Cl^−^, NO_3_^−^, and SO_4_^2−^) while it was deemed poor to unsuitable for heavy metals according to the water quality index (WQI). Among the carcinogenic constituents, As poses the greatest carcinogenic risk, particularly for children. The mean value of Cr, Mn, and As in the HQ_ingestion_ for adult and child, and Cd, Hg for child exceeded the threshold value established by the United States Environmental Protection Agency (USEPA), while the HQ_dermal_ values remained below the maximum limit for all heavy metals. The value of HI at all locations exceeds the threshold of 1, as specified by USEPA. Principal component analysis (PCA) and cluster analysis revealed that the presence of heavy metals in the Turag River was mainly attributed to anthropogenic sources, including industrial effluent discharge from neighboring industries, domestic wastewater, and agricultural runoff containing agrochemicals from the surrounding lands.

## Highlights

• HPI, P_N_ indicates high pollution & C_d_ indicates moderate to significant contamination.
• ERI and WQI indicates high risk due to elevated level of Hg.
• HI value exceeds the standard limit of 1 in both adults and children.
• Carcinogenic risk of As was greatest for child.
• PCA indicates the sources of heavy metals are mainly anthropogenic.

## Abbreviation

EC: -Electrical conductivity
TDS: -Total dissolved solids
DO: -Dissolved oxygen
COD: -Chemical oxygen demand
TH: -Total hardness
SAR: -Sodium adsorption ratio
MAR: -Magnesium adsorption ratio
Na: -Sodium
K: -Potassium
Ca: -Calcium
Mg: -Magnesium
Cl^−^: -Chloride
F^−^: -Fluoride
NO_3_^−^: -Nitrate
SO_4_^2^: -Sulfate
Pb: -Lead
Cd: -Cadmium
Cr: -Chromium
Ni: -Nickel
Fe: -Iron
Mn: -Manganese
Zn: -Zinc
Cu: -Copper
As: -Arsenic
Se: -Selenium
Hg: -Mercury
WHO: -World Health Organization
P_N_: -Nemerow pollution index
C_d_: -Contamination degree
HPI: -Heavy metal pollution index
ERI: -Ecological risk index
WQI: -Water quality index
HQ: -Hazard Quotient
USEPA: -United State Environmental Protection Agency
HI: -Health Hazard Index
PCA: -Principal component analysis
HRA: -Human health risk assessment
HCA: -Hierarchical cluster analysis
BSCIC: -Bangladesh Small and Cottage Industry Corporation
PET: -Polyethylene terephthalate
HNO_3_: -Nitric acid
APHA: -American Public Health Association
RSD: -Relative standard deviation
LOD: -Limit of detection
QL: -Quantification limit
ECR: -Environmental conservation rules
DPHE: -Department of public health engineering
FAO: -Food and Agriculture Organization
RI: -Risk index
IARC: -International Agency for Research on Cancer
TCR: -Total carcinogenic risk
KMO: -Kaiser-Meyer-Olkin

## 1 Introduction

Water is a crucial element for the sustenance of all forms of life on our planet [1]. Nevertheless, it is concerning that approximately 780 million individuals globally do not have access to improved water sources, according to the World Health Organization (WHO) in 2014 [2]. The world's surface water quality is threatened as ecosystems are altered and pollutants are released into rivers [3]. River water is highly significant to humans due to its wide range of uses (domestic, industrial, and agricultural) and its value in terms of transportation, tourism, and recreation. It plays a crucial role in supporting livelihoods [4]. According to the World Health Organization (WHO) in 2014, approximately 187 million individuals depend on surface water as their primary source of drinking water. The reliance on river water has been growing due to the rise in population, rapid urbanization, and industrialization. The growing global demand for surface water to meet human needs will place significant pressure on natural resources and ecosystems [5]. The decline in the water quality of rivers is often associated with industrialization, as untreated or partially treated effluents are frequently discharged into the river [6,7]. Due to the negative impact of pollution and the increasing contamination of surface water, there is a growing concern about the future availability of clean and safe water for everyone [8]. Ensuring the provision of safe water is increasingly difficult in developing countries, as both ground and surface water sources are being contaminated due to the absence of adequate wastewater treatment and drainage infrastructure [9]. A significant portion of sewage in developing countries is released into water bodies without undergoing any treatment, as reported by the UN-Water Decade Programme on Advocacy and Communication (UNWDPAC) in 2012 [10]. Approximately 1.8 million individuals in developing countries lose their lives each year due to water-related diseases [11]. In Bangladesh, water, sanitation, and hygiene-related issues contribute to approximately 8.5 % of deaths [12]. Residing in proximity to the contaminated rivers has been linked to an increased susceptibility to waterborne illnesses. Specifically, the communities located near the rivers in Dhaka city were found to have a higher risk of typhoid infection compared to other areas [13]. This is due to their dependence on contaminated surface water for various activities such as drinking, cooking, bathing, and more [13,14]. Given the various obstacles, achieving enhanced water quality on a global scale by 2030, through measures such as pollution reduction, elimination of dumping, and treatment of wastewater, is acknowledged as a significant objective within the United Nations Sustainable Development Goals [15].

Bangladesh, as a nation with a riverine landscape, is encompassed by numerous rivers. The process of rapid urbanization and industrialization has led to the contamination of river water systems with heavy metals and various other pollutants [16]. The primary contributors to water contamination are environmental pollution resulting from municipal, industrial, and agricultural wastewaters, as well as landfill leachate and runoff from urban areas and farmlands [17]. Water contamination caused by heavy metals poses a significant risk to human health due to their long-lasting and harmful effects [18]. Not all heavy metals have detrimental effects on the environment. Zn, Cu, Mn, and Fe are metabolically important among the heavy metals, whereas at low concentrations, non-essential heavy metals like Cd, Ni, Hg, Pb, Cr, and As can harm humans even in small amounts [[19], [20], [21]]. Industrialization is attributed to river water quality degradation due to the discharge of untreated or partially treated effluents [3,6,7]. The alteration of ecosystems and the discharge of contaminants into rivers pose a threat to the global water supply security as river water quality plays a critical role for ecosystems and human health [3]. The river water in Dhaka, Bangladesh, is polluted by untreated wastes from various industries, including textile, tannery, metal, dyeing, pharmaceutical, chemical, paper, frozen food, paint, and battery [16,[22], [23], [24], [25], [26]]. The textile industry generated 217 million cubic meters of wastewater in 2016 which was discharged into the river water system [22,23,25,26]. The Turag-Tongi-Balu system experiences high levels of contamination throughout the year due to various sources [27,28]. The water quality of the Turag River is negatively impacted by insufficient river management and uncontrolled industrial expansion [22,27]. Turag River is polluted by various industries including tanneries, garment manufacturers, metal and dyeing industries, pharmaceutical and chemical industries, paper factories, frozen food companies, paint factories, and battery factories [24]. Extensive research indicates that the rapid expansion of urban areas has had detrimental effects on the Turag River ecosystem and has led to a decline in the city's water resources [[29], [30], [31], [32]]. Health risks may arise when edible crops are irrigated with polluted river water, as the metals present in the water may accumulate in the soil and then be taken up by the plants cultivated in it [[33], [34], [35]].

Numerous investigations have been carried out to evaluate the Turag River water quality through the measurement of different physicochemical parameters [24,25,32,[36], [37], [38]]. A comprehensive study on water quality evaluation using a multi-index analysis approach has not been done yet on Turag River in Bangladesh. The rationale behind this work stems from the observation that while numerous studies have been conducted to evaluate the quality of Turag river water using various physical and chemical parameters, there remains a gap in research regarding the application of a multi-index analysis approach to comprehensively assess the ecological and human health risks associated with water quality in this context. The main objectives of this study are: (1) to investigate physicochemical parameters as well as current concentration of heavy metals in Turag River; (2) to demonstrate the pollution level in water through multi indices such as Heavy Metals Pollution Index (HPI), Contamination Degree (C_d_), Nemerow Pollution Index (P_N_), Water Quality Index (WQI); (3) to assess ecological risk index (ERI) and human health risk assessment (HRA) from metals; and (4) to find out the possible sources of these metals through PCA and HCA. The analysis of the collected data sheds light on the influence of heavy metals on water contamination and explores the underlying factors contributing to this issue. By bringing attention to the problem of water pollution in the Turag River, the study seeks to increase public awareness and inspire action from relevant authorities. Ultimately, the preservation of river water quality is emphasized as crucial for the well-being of aquatic ecosystems and human health, highlighting the urgency of addressing water pollution issues in the Turag River and beyond.

## 2 Materials and methods

### 2.1 Study area

**Fig. 1:**
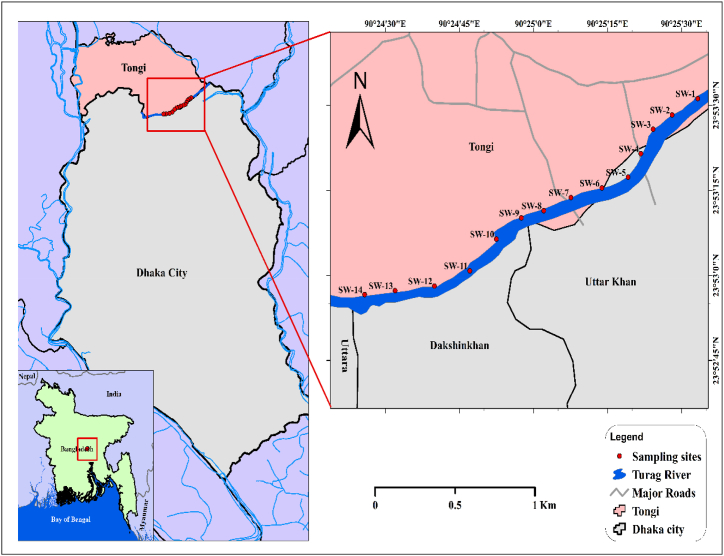
Map showing the Turag River of Dhaka, Bangladesh with sampling points.

Turag River, a significant upper tributary of the Buriganga river, is considered to be one of the major rivers in Dhaka, Bangladesh. The range of sampling locations spans from 23°53′31.2ʺ N to 23°52′56.6ʺ N latitude and 90°25′33.3ʺ E to 90°24′25.8ʺ E longitude. The sampling locations are presented in Fig. 1. The fourteen designated sampling locations extended from the Tongi BSCIC industrial zone to Nouka ghat, Abdullahpur, Uttara. A control sample was obtained near Nouka ghat, characterized by the absence of industrial activities.

### 2.2 Water sample collection and processing

Water was sampled at fourteen places near outflow points, waste dumping areas, plastic disposal areas, and areas where plastic is often washed during the dry season. The water sample was taken in 1000 mL PET bottles washed with 2 % HNO_3_. The PET bottles underwent three rinses with river water prior to sampling. Three water samples from each sampling station were taken for physiochemical and heavy metal analysis. A portion of each sample was separated for metal analysis and acidified with 2–3 drops of analytical reagent-grade concentrated HNO_3_ to attain a pH level of less than 2 [39]. The bottles were sealed, labeled, brought to the lab, and refrigerated until analysis. For metal analysis, 100 mL of acid-preserved water sample was digested with concentrated HNO_3_ [40]. After cooling to room temperature, it was then diluted to 100 mL with deionized water and then filtered through Whatman No. 42 filter paper. After digestion, samples were refrigerated at 4 °C for chemical analysis [39,41].

### 2.3 Sample analysis and methods

The pH, EC, DO and TDS was estimated at the field by using multimeter (SenIon156, HACH, USA). COD was measured according to APHA Standard Method 5220D (Spectrometer DR3900, HACH, USA). TH of water was calculated based on the individual measurements of Ca and Mg, according to APHA standard method 2340B which is as follows [41]:

(1) Hardness (mg equivalent CaCO_3_/L) = 2.497 [Ca, mg/L] + 4.118 [Mg, mg/L]

The SAR value was determined using the following equation

(2) SAR = [Na^+^] ÷ (√½ {[Ca^2+^] + [Mg^2+^]})

where the concentrations of all ions were expressed in milliequivalents per liter [42].

The calculation of the MAR was performed using the following equation:

(3) MAR = {Mg^2+^/(Mg^2+^+Ca^2+^)} × 100

where, the concentrations of all ions were given in meqL^−1^ [43].

The quantification of major cations, such as Ca^2+^ and Mg^2+^ was conducted using an Atomic Absorption Spectrophotometer (Shimadzu, AA-7000, Japan). Na^+^ and K^+^ were estimated by Flame Photometer (PFP 7, Jenway, UK). The estimation of the major anions was conducted by using the Ion Chromatograph (Dionex, ICS-1600, Thermo Scientific, USA) according to the standard procedure [39]. An inductively coupled plasma mass spectrometry (ICP-MS) (PerkinElmer, NexION 2000, USA) was employed to analyze heavy metals in the water samples. The data obtained from the ICP-MS analysis was analyzed using the Syngistix™ software to determine the amounts of heavy metals contained in the samples [44].

### 2.4 Quality assurance and quality control

The glassware used in this investigation was meticulously cleaned with ultra-pure acid and water to reduce contamination during sample preparation and analysis. Before calibrating the ICPMS equipment with standards, NexION Setup Solution (PerkinElmer, USA) was used to evaluate its performance. A successful calibration variance of five points was achieved, with an R^2^ value over 0.9995, spanning 1.0–20.0 μg/L. To ensure equipment reliability, three replications were done. RSD was allowed between 5 % and 10 %. The instrument's LOD and LOQ were calculated, determining the threshold for a metal signal over background noise. The recovery percentage of specific elements was assessed using river water certified reference material SLRS-6, with a recovery range of 95–105.2 % for significant elements (Table 4). There was no certified value for Se and Hg in the reference material, SLRS-6. All the metals were measured in triplicate and are given as mean value in μg/L. Quality control measures included method installation, SRM recovery evaluation, and spike recovery analysis to minimize errors. Trace metal quantities were adjusted based on recoveries and blank sample analyses [45,46].

### 2.5 Data interpretation methods

#### 2.5.1 Heavy metals pollution index (HPI)

The study presents an equation representing the HPI, a crucial tool for evaluating water quality based on the collective impact of individual trace metals, derived from weighted arithmetic quality means [[47], [48], [49], [50], [51]]:

(4) $$HPI=\left(\sum_{i=1\;}^{n}WiQi\right)/(\sum_{i=1}^{n}Wi$$

Wi is the assigned weightage to every trace metal; N represents the total quantity of metals that have been assessed.

Qi is the quality rating of ith metal given as:

(5) $$\text{Qi}=\sum_{i=1}^{n}\frac{Mi-I}{Si-I}x100$$

Mi is the measured value of the ith metal in parts per billion (ppb), Si represent the standard concentration of each metal, and I is the ideal value of each metal in water.

#### 2.5.2 Contamination degree (C_d_)

The C_d_ is a metric that measures the contamination level of each metal and the cumulative impact of all metals collectively, categorizing contamination into low, medium, and high levels using formula as follows [47,52]:

(6) $$C_{d}=\sum_{i=1}^{n}Cfi$$

(7) $$Cfi=\left(\frac{CAi}{CNi}\right)$$

Cfi is the contamination factor of ith metal, CAi is the measured value for ith metal, and CNi is the maximum permissible limit of ith metal

#### **2.5.3** **Nemerow pollution index (**P_N_)

The Nemerow pollution index (P_N_) technique emphasizes the most polluting element while also considering different factors [53]. This method allows for thorough heavy metal contamination assessment at particular sites, taking into consideration diverse heavy metals' effects. P_N_ can be calculated by

(8) $$P_{N}=\left(\sqrt{P_{i}^{2\;}+P_{i\left(\max\right)}^{2}}\right)/2$$

Where, $P_{i}^{2}$ represents the arithmetic mean of the pollution index for all pollutants and $P_{i\left(\max\right)}^{2}$ stands for the maximum pollution index observed across all pollutants, calculated by considering the individual pollution index at each site.

#### 2.5.4 Ecological risk index (ERI)

The ecological risk index (ERI) and risk index (RI) was used to quantify risk by comparing pollutant toxicity to background value by using Hakanson's equation [54]. The following equations calculate ERI and RI:

(9) $$E_{r}^{i}=T_{r}^{i}\times C_{f}^{i}$$

(10) $$RI=\sum_{i=0}^{n}E_{r}^{i}$$

Where $C_{f}^{i}$ represents the contamination factor associated with a particular element ‘‘i’’; $T_{r}^{i}$ denotes the toxic-response factor associated with a particular element ‘‘i’’. The toxic-response factors for Pb, Cd, Cr, Cu, Zn, As, Mn, Hg were determined to be 5, 30, 2, 5, 1, 10, 1, and 40 accordingly [54,55].

#### 2.5.5 Water quality index (WQI)

The water quality index (WQI) measures how different factors affect water quality [56]. The WQI was calculated using the following equation:

(11) WQI=∑QnWn/∑Wn

Where, Qn = quality grading assigned to each water quality parameter, Wn = weight of units for each indicator of water quality

(12) Qualityrating(Qn):Qn=[(Vn−Vid)/(Sn−Vid)]X100

Where, Vn = estimated each water quality parameter value, Vid = ideal parameter values in pure water, Sn= Standard acceptable limit for each water quality parameter.

(13) Unitweight(Wn):Wn=k/Sn

Where, Sn = Standard acceptable limit for each water quality parameter

k = constant proportionality, determined by utilizing the equation provided below:

(14) $$k=\left[1/(\sum \left(\frac{1}{Sn}\right)\right]$$

#### 2.5.6 Human health risk assessment (HRA)

Human health risk due to the exposure of trace metals through the well-known pathways of ingestion and dermal contact [[57], [58], [59]] can be assessed by using the formula developed by USEPA [[60], [61], [62], [63]].

(15) $${CDI}_{ingestion}=\frac{\text{EC}\times \text{Ing}\;R\times \text{EF}\times \text{ED}}{\text{BW}\times \text{AT}}$$

(16) $${CDI}_{dermal}=\frac{\text{EC}\times \text{SA}\times \text{AF}\times \text{ABS}\times \text{ET}\times \text{EF}\times \text{ED}\times \text{CF}\;}{\text{BW}\times \text{AT}}$$

where the measured concentration of metals in mg/L is denoted by EC; IngR is the ingestion rate of water in L/day i.e. 2.5L/day for adult and 0.78L/day for child [60]; EF is the exposure frequency in day/year i.e. 365 [60]; ED is the exposure duration in year which is 70 for adult and 6 for child [64]; BW is the average bodyweight of adult (70 kg) and child (20 kg) [65]; AT is the average time in a day (ED X 365) [60]; SA is the exposed skin area in cm^2^ (18000 cm^2^ for adult and 6600 cm^2^ for child) [64,66]; AF is the adherence factor (mg cm^2^) (0.07) [66]; ABS is the dermal absorption fraction (0.03) [66]; the exposure time (ET) is 0.6 (hour day^−1^) [67]; CF is the conversion factor (kg/mg) (10^−6^) [64].

The hazard quotient (HQ) is a method employed to assess the non-carcinogenic risk associated with trace metals in water intake, using the following formula [23,68]:

(17) $${HQ}_{ingestion}=\frac{\text{CDI}\;\text{ingestion}}{\text{RfD}\;\text{ingestion}}$$

(18) $${HQ}_{dermal}=\frac{\text{CDI}\;\text{dermal}}{\text{RfD}\;\text{dermal}}$$

(19) $$HI=\sum {HQ}_{i}$$

where i denotes the HQ of each trace metal, RfD ingestion and RfD dermal are the reference dose for oral and dermal exposure in mg/kg/day which stands 0.0014, 0.0005, 0.003, 0.02, 0.04, 0.3, 0.02, 0.0003, and 0.0003 for ingestion and 0.00042, 0.000005, 0.000015, 0.0054, 0.012, 0.06, 0.0008, 0.0003, and 0.0003 for skin contact in terms of Pb, Cd, Cr. Ni, Cu, Zn, Mn, As, and Hg respectively [65,[69], [70], [71]]. Carcinogenic risk assessment of population group results from the exposure of potential carcinogens i.e. a group of metals that have a particular cancer slope factor (CSF) over the whole period of life [[72], [73], [74], [75]]. The Carcinogenic risk of an individual metal (Carcinogenic risk i) and numerous metals (Total carcinogenic risk) were measured using the following equations:

(20) $${Carcinogenic\;risk}_{i}=CDI\times SF$$

(21) $$Total\;Carcinogenic\;risk=\sum_{i=1}^{m}\sum_{j=1}^{n}Risk\;ij$$

Where, Carcinogenic risk for an individual is calculated by chronic daily intake of metals through ingestion and dermal absorption using the slope factor of individual metals. The applied slope factors (mg/kg/day) were 1.5, 0.0085, 0.5, and 15 for As, Pb, Cr, and Cd respectively. Total carcinogenic risk is the cumulative cancer risk associated with each heavy metal (i) in the various exposure pathways (j).

### 2.6 Multivariate statistical analysis

Principal component analysis (PCA) linearized source data to decrease large groups of variables. Linear combinations provide orthogonal, uncorrelated variables that maintain most of the dataset details. The primary variable covariance matrix can be used to calculate eigenvalues and eigenvectors [76]. Analyses were conducted to determine the primary causes of heavy metals in surface water samples and their sources and fluctuations. The Pearson correlation matrix shows what variables are related. The hierarchical cluster analysis (HCA) was used to find out the similarity among sampling sites and among variables. The correlation coefficient matrix was used to measure how much each component's variance is explained by its relationships [77]. This study statistically analyzed experimental river water data using IBM SPSS (Version: 20) and Minitab (Version: 17.0). Representative figures were illustrated by GraphPad Prism (v. 8), origin pro (v. 9.0).

## 3 Results and discussion

### 3.1 Physicochemical properties of river water

**Fig. 2:**
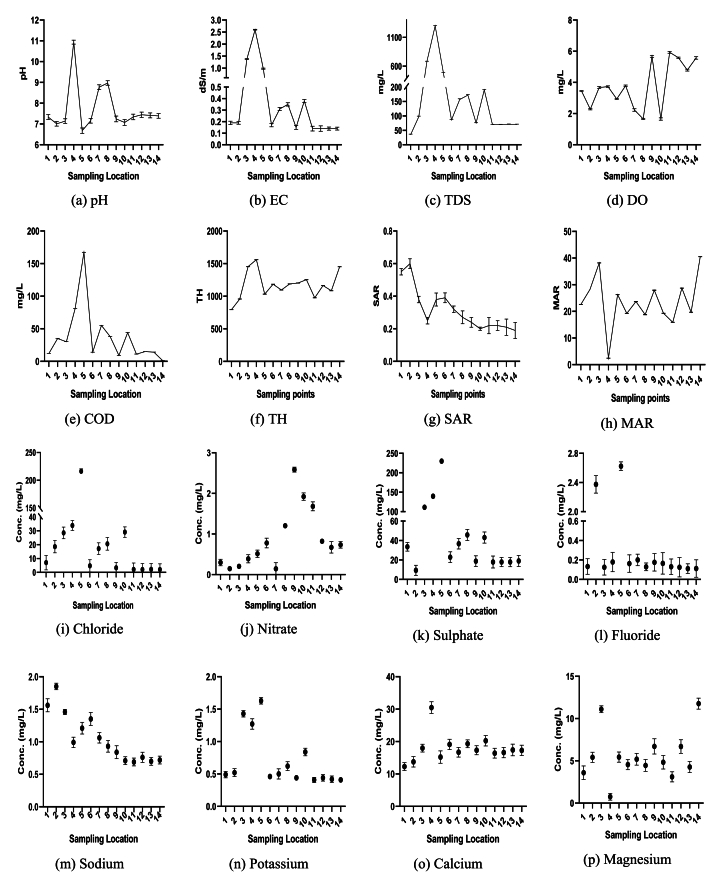
Spatial variations in the physicochemical properties of the Turag River water at different sampling points: (a) pH, (b) EC, (c) TDS, (d) DO, (e) COD, (f) TH, (g) SAR, (h) MAR; concentrations of anions including (i) Chloride, (j) Nitrate, (k) Sulfate, (l) Fluoride; and concentrations of cations including (m) Sodium, (n) Potassium, (o) Calcium, and (p) Magnesium.

The physicochemical properties of Turag River water from different sites is illustrated in Fig. 2. The pH values ranged from 7.01 to 10.94, with a mean of 7.71 ± 1.13 (Table S1 and Fig. 2a). Sampling point S4, located near a metal alloy industry, exhibited the highest pH of 10.94, exceeding the standard value of 8.5 [69,82], while sampling point S5, situated near a shared drainage system, had the lowest pH value. These pH fluctuations are attributed to waste discharge from various sources, including industrial and household activities, as well as routine practices such as cleaning, bathing, and toileting near the river. These factors have also been documented in other studies [[78], [79], [80]]. While numerous fish species can adapt to a wide pH range of 5.0–9.0, variations in pH levels significantly affect the solubility and availability of chemicals, leading to nutritional challenges for aquatic species [78,79].

The comprehensive quantification of dissolved salts and ionic compounds in water is achieved by assessing electrical conductivity (EC), where elevated EC levels often indicate higher salinity and mineral content, impacting both the ecology of surface water and its suitability for various uses [81]. The EC values of water samples from different locations ranged from 140.3 to 2600 μS/cm, with an average of 518.39 ± 701.56 μS/cm (Table S1 and Fig. 2b). Sample point S4, located near the metal alloy industry, exhibited the highest EC at 2600 μS/cm, significantly exceeding the standard permissible limit of 300 μS/cm [69,82]. In contrast, sampling point S11, situated in an area with no notable industrial or domestic activities, had the lowest EC at 140.3 μS/cm. The elevated EC levels are attributed to the release of industrial and domestic waste, along with activities such as cleaning, bathing, and toileting near the river. These factors have been documented in several other studies [[78], [79], [80]]. High EC can disrupt the internal fluid balance of salinity-sensitive aquatic organisms, causing stress or mortality, and negatively affect fish reproduction and growth. With the exception of areas S3 and S4, EC values were below the WHO limit of 80–1000 μS/cm [69,82].

The TDS levels in the samples ranged from 34.9 to 1300 mg/L (Table S1 and Fig. 2c), with an average of 254.89 ± 353.24 mg/L. Sampling location S4, located near the metal alloy industry had the highest TDS value at 1300 mg/L which exceed the permissible limit of 1000 [69,82,86]. In contrast, sampling point S1, located near the waste dumping area, had the lowest TDS value. The changes in TDS occurred due to the industrial and domestic waste discharge from the surroundings of the sampling points. These factors have also been noted in several other studies [[78], [79], [80]]. TDS has a notable impact on water temperature, photosynthesis, water clarity, and the well-being of aquatic habitats and creatures. Higher levels of TDS can have a negative impact on the habitats of aquatic organisms and reduce biodiversity [83]. Nevertheless, the TDS levels measured at various locations were significantly below the WHO's recommended threshold of 1000 mg/L, except for S4, which has been specifically designated to protect fisheries, aquatic organisms, and residential water sources [69,82].

The DO in water is crucial for evaluating the oxygen levels necessary to sustain a thriving and diverse aquatic ecosystem. Low DO levels often indicate poor water quality, making DO monitoring vital for detecting pollution and evaluating the overall health of rivers [84]. Low DO levels are typically indicative of poor water quality. The study found that DO levels varied between 1.65 and 5.93 mg/L, with an average of 3.78 ± 1.52 mg/L (Table S1 and Fig. 2d). Various factors, including pollution, excessive organic matter (eutrophication), and high temperatures, lead to a depletion of dissolved oxygen (DO) levels in the studied areas. The impact of these factors has also been observed in various other studies [[78], [79], [80]]. In the studied areas, DO levels were found significantly lower than the recommendations value set by the Food and Agriculture Organization (FAO) [85]. Out of the sample sites S14, located within the boat wharf areas, had the highest DO level of 5.93 which was close to the standard value of 6 [86], whereas sampling point near the agricultural land, S10 had the lowest DO level.

COD is a key indicator of organic and inorganic contaminants in water bodies, particularly in rivers. Elevated COD levels can result in lowered oxygen levels in the water, which have a detrimental effect on aquatic organisms as high COD levels lead to increased oxygen consumption during the oxidation process. The levels of COD in the water samples varied from 1.0 to 167 mg/L (Table S1 and Fig. 2e). The results indicated that S5 had the highest COD, while S14 had the lowest. The average value of COD was found 37.57 ± 43.13 mg/L, which falls within the acceptable limits of 50 mg/L [69,82,86], except for sites S4, S5, and S7 which has COD values of 81, 167, and 55 mg/L respectively. These sites were near the outlet areas of industrial processes. The higher value of COD occurred due to high levels of decaying plant matter, human waste, or industrial effluents as numerous additional research have also noted the influence of these factors [[78], [79], [80]].

Total hardness (TH) is a critical water quality metric in rivers and freshwater bodies, measuring the concentration of calcium and magnesium ions in terms of CaCO_3_ equivalents. Excessive hardness can affect water's taste, appearance, and suitability for various uses [87,88]. Changes in water pH and chemistry due to high TH can also threaten aquatic life [89,90]. In this study, TH was calculated using equation (1), revealing a range from 793.90 to 1454 mg/L, with an average of 1171.49 ± 210.87 mg/L (Table S1 and Fig. 2f). The highest TH value of 1454 mg/L was recorded at location S3 near the municipal discharge outlet and agricultural land while the lowest value of 793.90 mg/L was observed at location S1 near the waste dumping area, both exceeding the standard limit of 300 mg/L [69,82,86]. The elevated hardness is attributed to increased levels of calcium and magnesium in water, consistent with findings of several other studies [[78], [79], [80]].

Water SAR, calculated from the concentrations of sodium, calcium, and magnesium using equation (2), is a crucial indicator for water use in agriculture and aquaculture. In this study, SAR values ranged from 0.19 to 0.60 (meq/L), with a mean of 0.32 ± 0.13 (meq/L) (Table S1 and Fig. 2g), significantly lower than the permissible limit of 9 (meq/L) [69,82]. High levels of harmful substances can harm cells and disrupt important functions like breathing and salt regulation [87]. Using irrigation water with a high SAR for a long time can result in sodium ions replacing calcium and magnesium in the soil. This can have a negative impact on the formation of stable soil aggregates, leading to poor soil structure and tilth. This can lead to a decrease in soil permeability and infiltration, resulting in lower crop productivity. The water in the studied regions is suitable for irrigation and aquaculture, as its SAR values are within the acceptable range.

The theoretical framework of MAR examines sodium-induced cation exchange in soil, influenced by the presence of magnesium and other cations in water. When evaluating water appropriateness for irrigation and its possible impacts on soil composition and plant development, the MAR approach is essential in the crop cultivation sector. Equation (3) was used to compute MAR using the calcium and magnesium values. According to Table S1 and Fig. 2h, the study found that MAR values ranged from 2.43 % to 40 %, with an average of 23.67 ± 9.44 %. These results show that the water in the investigated areas is appropriate for both irrigation and aquaculture since they are within the permissible range of 50 % [69,82].

### 3.2 Concentration of anions and cations in the Turag River water

Water samples were examined using ion chromatography to determine the presence of anions. The utilization of river water for domestic and/or agricultural purposes is hindered as a result of the heigh content of diverse dissolved salts [81]. The concentrations of anions and cations are given in Fig. 2(i–p), Tables S2 and S3. The results of the analysis indicated the presence and quantification of fluoride (F^−^), chloride (Cl^−^), nitrates (NO_3_^−^), and sulfate (SO_4_^2−^) anions in the water samples collected from the studied locations. However, bromide (Br^−^), nitrite (NO_2_^−^), and phosphate (PO_4_^3−^) were quantified below the method detection limit (MDL). The MDL for these anions was determined to be 0.050 mg/L for bromide, 0.025 mg/L for nitrite, and 0.045 mg/L for phosphate, respectively.

The chloride (Cl^−^) levels in the water of the studied areas varied significantly, ranging from 2.07 to 216.27 mg/L, with an average value of 27.66 ± 55.49 mg/L (Fig. 2-i, Table S2). Out of all the locations, S5, near the outlet of industrial process had the highest chloride value while S14, within the boat wharf areas had the lowest. However, the concentration of chloride in all locations remained within the standard limit of 250 mg/L [69,82]. Elevated chloride concentrations in river water signify pollution and are commonly attributed to sources such as road salt, industrial discharge, wastewater, and urban runoff. Numerous studies have documented the significant impact of these factors on chloride levels in aquatic environments [80,91].

The nitrate (NO_3_^−^) concentrations across the different sampling locations ranged from 0.1432 to 2.5861 mg/L, with a mean value of 0.86 ± 0.74 mg/L (Fig. 2-j, Table S2). The highest nitrate concentration was observed at sampling point S9, in the zone of plastic washing while the lowest was at S7 near the outlet of industrial process. The nitrate levels in all sampling locations were within the acceptable limit of 50 mg/L [69,82]. The presence of nitrates in the river water was attributed to agricultural runoff and sewage discharge, consistent with findings from other studies [91]. Elevated nitrate levels can lead to eutrophication, where an excess of nutrients stimulates overgrowth of plants, particularly algae. This overgrowth depletes oxygen in the water, adversely affecting aquatic ecosystems [91].

The amounts of sulfate (SO_4_^2−^) in the water across the locations under investigation varied significantly, with an average of 54.52 ± 63.14 mg/L and a range of 9.31–229.93 mg/L (Fig. 2-k, Table S2). sample location S5, which was close to industrial discharge outlets, had the highest concentration, whereas sample point S2 had the lowest concentration. All sulfate levels were within the permissible limit of 250 mg/L [69,82]. The sulfate concentrations primarily originated from anthropogenic sources, including industrial processes and agricultural runoff, corroborating findings from another research [91]. Sulfate can combine with water to form sulfuric acid, leading to acidification and posing a threat to aquatic life in the river [91].

The findings revealed that the fluoride (F^−^) concentrations across the different sampling locations ranged from 0.1247 to 2.3742 mg/L with mean value of 0.48 ± 0.86 mg/L (Fig. 2-l, Table S2). The highest value was observed at sampling location S2, close to outlets of industrial processes and lowest was at S3 which located close to municipal waste discharge outlets and agricultural land. Fluoride is a naturally occurring substance in river water, albeit the amount varies depending on the chemistry of the surrounding water, and human activities [91]. Although the amounts of fluoride in the studied areas were found within the permissible limit of 1.5 mg/L [69,82], but it can be affected by the human activities such as runoff from agricultural land and industries which was consistent with findings of another study [91].

A comprehensive description of the presence of different anions along with their corresponding concentrations are listed in Table S2 and Fig. 2i-l. This study quantified numerous anions in descending order: chloride > sulfate > nitrate > fluoride. In case of fluoride, except for location S5 and S2, the concentrations were found within the recommended safe limit proposed by the World Health Organization in 2004 and 2006 [69,82]. Moreover, by comparing the standard, the concentrations of chloride, sulfate and nitrate were also found within the permissible limits [69,82]. The status of different anions in Turag River water is mainly influenced by activities like industrial processes, agricultural runoff, washing plastic bags, and dumping of waste at close proximity of the river [91].

The findings of the cationic test, specifically the levels of Na, K, Ca, and Mg, are presented in Table S3 and Fig. 2m-p. The results suggest that the concentrations of sodium (Na), potassium (K), calcium (Ca), and magnesium (Mg) in the collected samples were within the established reference ranges [69,82,92]. The analysis revealed that the levels of sodium (Na), potassium (K), calcium (Ca), and magnesium (Mg) in the water samples obtained from various points along the Turag River ranged from 0.69 to 1.85 mg/L, 0.41–1.43 mg/L, 12.29–30.47 mg/L, and 0.76–11.77 mg/L, respectively. The highest value of these cations were found within the standard permissible limits [69,82,92].

Based on the comprehensive analysis of anionic and cationic concentrations, it can be concluded that the Turag River does not exhibit significant signs of pollution. The occurrence of calcium (Ca) and magnesium (Mg) in the river water can be ascribed to their inherent prevalence in the Earth's crust. Calcium and magnesium can enter river systems through the weathering of rocks and soil, contributing to the observed levels in the water samples. Usually, the calcium levels in river water are found in relatively low concentrations, ranging from 1 to 2 mg/L [81].

### 3.3 Heavy metals contamination in the Turag River water

**Fig. 3:**
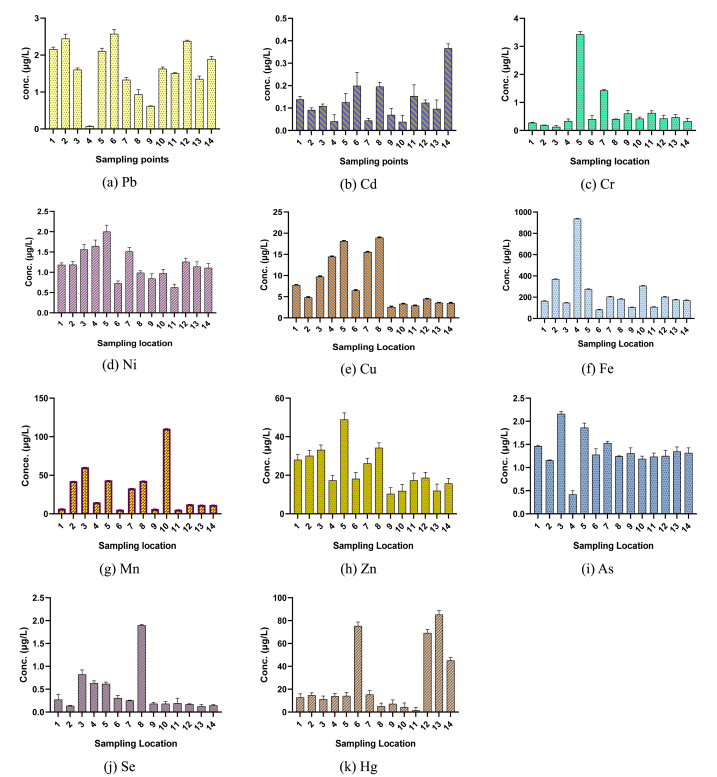
Spatial variation of trace metal concentrations in the Turag River water at different locations: (a) Pb, (b) Cd, (c) Cr, (d) Ni, (e) Cu, (f) Fe, (g) Mn, (h) Zn, (i) As, (j) Se, and (k) Hg.

The concentrations of heavy metals (Pb, Cd, Cr, Ni, Cu, Fe, Mn, Zn, As, Se, and Hg) in water samples from the studied areas are presented in Table S4 and Fig. 3a-k. Industrial and anthropogenic activities in urban areas significantly influence the metal concentrations in the river water. The concentrations of these metals were assessed against standard acceptable limits [69,70,82,85,86]. Table 2 presents a comparative analysis of the heavy metal concentrations in the water of the Turag River, in contrast with results obtained from various studies conducted in Bangladesh and abroad. The concentrations of heavy metals (Pb, Cd, Cr, Ni, Cu, Fe, Mn, Zn, As, Se, and Hg) in water samples of the studied areas are shown in Table S4 and Fig. 3a-k. The concentration of metals in river water are affected by industrial and anthropogenic activities in urban areas. The levels of these metals were evaluated using standard acceptable limits [69,70,82,85,86]. A comparison of the heavy metal concentrations in the Turag River water with those found in various other studies conducted in Bangladesh is shown in Table 2.

The highest concentration of lead (Pb) was observed at the S6 location, near the industrial discharge outlet areas, with a concentration of 2.572 μg/L. The lowest level of Pb was found at the S4 location, which is situated near both industrial activities and the designated bathing area, with a concentration of 0.071 μg/L (Table S4). The concentration of Pb in the studied areas ranged from 0.071 μg/L to 2.572 μg/L with an average of 0.61 ± 0.72 μg/L (Table S4 and Fig. 3a), which were significantly lower than the standard limit of 50 μg/L [69,82]. The sources of lead in water attributed from the industrial process like metal plating, runoff of agrochemicals from neighboring lands, industrial effluent discharge and automotive exhaust. The observed concentrations of Pb in the study region was much lower than the previous studies which stated that the value of Pb was 16 μg/L and 65.45 μg/L in Shitalakhya and Buriganga river respectively [84,93] and in Korotoa river the content of Pb was of 35.0 μg/L in winter and 27.0 μg/L in summer [94]. Another report provides justification for the sources of lead in Turag's river water [95]. Chronic Pb intoxication causes malignancies, anemia, reproductive harm in males, hormonal anomalies, and IQ reduction in early newborns [96,97].

This investigation revealed that Cd levels ranged from 0.04 to 0.37 μg/L (Table S4 and Fig. 3b), with a mean of 0.13 ± 0.09 μg/L. The highest level of Cd was found at S14 locations near the waste dumping places close to boat wharf areas and lowest was recorded at S4, location designated for bathing near the metal alloy industry (Table S4). Cd levels were found significantly lower than the standard limit of 10 μg/L at all monitoring locations [69,82]. The main contributors of Cd in the river water were the discharge of waste from the metal industry, coal combustion, and waste disposal sites. The findings on the Cd content in this study area was found much lower than the other investigation conducted on the other rives which reveals that the content of Cd in Korotoa river was 11 μg/L in winter and 8 μg/L in summer [93], 9.34 μg/L and 3 μg/L in Buriganga and Shitalakhya river respectively [19,94]. The findings of the present study align with the previous study [98]. Cd exposure causes calcium excretion and bone demineralization and the risk of bone fragility and fractures increases with increasing concentrations of Cd in water [99]. Cd exposure during pregnancy lowers birth weight and increases premature death risk [100].

Cr levels ranged from 0.18 to 3.43 μg/L, with an average of 0.67 ± 0.85 μg/L (Table S4 and Fig. 3c). The highest level of Cr was found at S5 locations near the industrial effluent discharge areas and lowest was recorded at S3 location of Turag River near the municipal waste dumping areas (Table S4). The result in all monitoring locations were found substantially lower than the maximum allowable limit of 100 μg/L [70]. The presence of Cr in water was determined to be caused by several industrial activities, including leather tanning, dyeing, chrome plating, industrial welding, and wood preservation, which are conducted along the Turag River. Additionally, the discharge of industrial waste into the river further contributes to the contamination. This finding is supported by previous investigations [101,102]. The comparable results were also found on the Korotoa River [93]. A high Cr level of 587.44 μg/L was observed in Buriganga River [94] where in river Shitalakhya the value was 18 μg/L Cr [84]. Cr is a well known carcinogen and should be removed from water prior to consumption or other uses [103].

The analysis found Ni content in the water ranging from 2.002 to 0.625 μg/L with a mean concentration of 1.20 ± 0.38 μg/L (Table S4 and Fig. 3d), substantially lower than the established permissible limit [85]. The location S11, which was mostly devoid of industrial, agricultural, and municipal activity, had the lowest Ni level, whereas the S5 location closest to industrial effluent discharge area and near the place of garbage burning, had the highest Ni levels (Table S4). The levels of Ni in water bodies can be attributed to the discharge of waste from power stations, steel factories, and garbage burning activities conducted along the studied areas in Turag River. A previous study aligns with the sources of Ni in water and reported a Ni concentration of 14 mg/L in river water [101].

The Cu content ranged from 2.55 to 18.93 μg/L, with an average of 8.28 ± 5.99 μg/L (Table S4 and Fig. 3e). The highest concentration of Cu was detected at S8 locations adjacent to agricultural land regions and in front of the Bangladesh Inland Water Transport Authority (BIWTA) office in Mirashpara, Tongi. Conversely, the lowest concentration was seen at the S9 location, an area characterized by the washing of used plastic sheets, bags, and other plastic items (Table S4). The presence of Cu in river water can be attributed to agricultural activities, agrochemical firms, and the discharge of urban sewage in the examined sections of the Turag River which is supported with a prior study [93]. The status of Cu in river water is important as Cu overdose can cause organ damage, vomiting, nausea, renal dysfunction, hemolytic jaundice, and central nervous system depression [104].

The Fe concentrations ranged from 81.76 to 936.30 μg/L, with a mean of 244.72 ± 214.35 μg/L (Table S4 and Fig. 3f), below the established permissible limit of 1000 μg/L [86]. The Fe concentration was highest at S4 location, adjacent to the metal alloy producing industry and characterized by the bathing of local peoples. The lowest concentration was found at sampling location S6, which was in close proximity to the industrial outlet areas. The presence of Fe in water is potentially attributed to the release of effluents from industries involved in the production of metal alloys. Moreover, it supports the conclusions of prior research indicating that the effluent from metal alloy manufacturers results in an overabundance of iron in water [104]. Excessive iron levels in the body can lead to iron toxicity, causing stomach and gastrointestinal issues [105].

The concentrations of Mn in the surveyed locations ranged from 5.29 to 110.273 μg/L with a mean value of 28.93 ± 29.64 μg/L (Table S4 Fig. 3g), was found below the standard permissible limit [85]. The site close to the industrial outlet, S6, had the lowest concentration of Mn, whereas the area near the agricultural field, S10, had the highest Mn content. The primary source of Mn in river water is attributed to the runoff of agrochemicals from agricultural land, as this element is naturally abundant in the Earth's crust. The previous research offers evidence supporting the origins of Mn concentration in river water [93]. The oxidation states of Mn in surface water are influenced by microbial activity and dissolved oxygen levels [106]. Exposure to manganese over a long period leads to anemia caused by iron deficiency and impairment of metabolism dependent on copper [107].

Zn levels in this study ranged from 10.30 to 48.88 μg/L, averaging 22.97 ± 10.93 μg/L (Table S4 and Fig. 3h) which was well below the permissible limit of 2000 μg/L [69,82]. The highest concentration of Zn was detected at location S5, which was in close proximity to the discharge region of industrial effluents. Additionally, burning of rubbish occurred near this site. Conversely, the lowest concentration of Zn was observed at position S9 (Table S4), location characterized by washing of used plastic sheets, bags, and other plastic items. The presence of Zn in the river water of the investigated regions is attributed to emissions from coal-fired power plants, steel manufacturing, and waste incineration near the river. These findings are well supported by another study [108]. In another study Zn content was observed 56 μg/L in Shitalakhya River water [101]. Normal physiology requires trace elements like Zn. However, high Zn concentrations may affect humans and aquatic life [109].

As concentrations in the water of the surveyed areas ranged from 0.42 to 2.16 μg/L, with a mean of 1.34 ± 0.39 μg/L (Table S4 and Fig. 3i) which were substantially lower than the recommended standard limit of 100 μg/L for surface water [86]. The highest level of As was detected at position S3, which was in close proximity to the municipal waste discharged outlet site and near to the agricultural land from where agrochemicals runoff may occurred. Conversely, the lowest concentration of As was found at S4 location characterized for bathing next to the metal alloy manufacturing industry. The sources of As in water is supported by other study which stated that As in river water derived from metal refining, industrial, fertilizer, and pesticide use and it can harm the liver and cardiovascular system of humans [110]. Several studies also found As in river water which stated that the Korotoa River had As levels of 46 μg/L in winter and 37 μg/L in summer [93] and the Shitalakhya River water had As contents below 10 μg/L [84].

Se concentrations in the studied samples ranged from 0.13 to 1.89 μg/L, with an average of 0.42 ± 0.48 μg/L (Table S4 and Fig. 3j), much below the maximum permissible limit of 20 μg/L [69,82]. Sample point S8, located near agricultural land areas and near the Bangladesh Inland Water Transport Authority (BIWTA) office at Tongi, exhibited the highest concentration of Se. Conversely, the lowest concentration was observed at S2, which was in close proximity to a solid waste dumping area (Table S4). Se accumulation in aquatic organisms, notably shellfish and fish, causes selenosis or poisoning. Elevated levels of Se can distort, hinder reproduction, and kill aquatic life. Human selenosis, hair and nail brittleness, and skin rashes can result from high Se levels [111,112].

Hg levels in water ranged from 1.36 to 22.73 μg/L (Table S4 and Fig. 3k), with an average of 8.23 μg/L exceeding the maximum permissible limit of 1 μg/L [69,84]. The concentration of Hg was found to be the lowest in sampling location S11, which exhibited minimal industrial, agricultural, and municipal activities. Conversely, the highest concentration of Hg was observed in S6, which was situated in close proximity to the industrial outlet area. Hg is introduced into river water systems through both natural sources, such as weathering, and human activities, including industrial processes and coal combustion. These findings align with a previous study [113]. The significance of Hg in river water lies in its potential to contaminate aquatic ecosystems, harm human health, and persist in the environment [113,114].

The findings of this study revealed that the concentrations of different heavy metals in Turag River water were detected in the following order: Fe > Mn > Zn > Cu > Hg > As > Ni > Cr > Pb > Se > Cd. Another study examining the heavy metal concentration of river water discovered that the highest observed values were for Zn and Mn in the Khoshk River, while Cd and Cu had the lowest values [115]. Previous studies have also identified industrial effluent, household sewerage, agricultural activities, and solid waste as significant contributors to contamination [69,82,116], which aligns with the findings of this study.

### 3.4 Index based assessment of pollution in river water

**Fig. 4:**
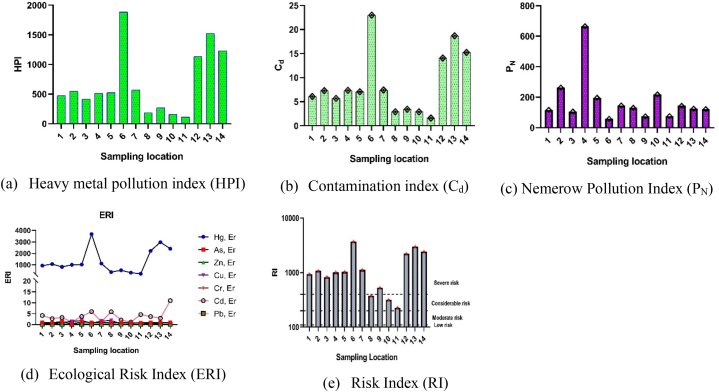
Distribution of trace metals pollution based on (a) HPI, (b) C_d_, (c) P_N_, and (d) ERI, and (e) RI in Turag River.

Indices are the indicators to environmental pollution caused by various contaminants which are intended to express the threat toward human as well as the ecosystem. The effect of metal contribution in water can be estimated from different pollution indicators such as heavy metal pollution index (HPI), contamination degree (C_d_), and nemerow pollution index (P_N_). The contamination degree (C_d_) is a versatile tool to identify the pollution and contamination level in environmental matrix. Contamination degree accounts for the degree of water contamination within a river due to the presence of contaminants like heavy metals as a whole. Heavy metal pollution index is a potential tool applied to evaluate the composite influence of heavy metals in water. Nemerow index is more comprehensive tool to find out the most polluting factor taking into consideration of other factors that reflects the water quality of each monitoring station. An ecological risk index provides a fast and simple quantitative value on the potential ecological risk of a given contamination situation in a river or lake or fresh water system. The distribution of trace metal pollution in the Turag River, assessed using HPI, C_d_, P_N_, ERI), and the overall risk index (RI), is illustrated in Fig. 4. The overall health status i.e. suitability or unsuitability was described by water quality index (WQI). These indices collectively contribute to a thorough and scientific evaluation of the Turag River's quality, enabling informed decision-making and targeted interventions for its preservation and restoration.

#### 3.4.1 Heavy metal pollution index (HPI)

The HPI was calculated using equations (4), (5)) for each element to assess pollution at fourteen sampling locations and the HPI values are shown in Fig. 4a. HPI values in the Turag River ranged from 112.85 to 1883.92 (Table S7). The average HPI index was calculated as 682.38 which surpasses the WHO limit of 100 for all sites [69,82]. All samples had HPI over the threshold value. S6 has the highest HPI and S11 had the lowest. Heavy metals discharged from fertilizer, pigment, paper, textile, and dirty plastic goods washing along the Turag River may contribute to these high pollution index values. The result revealed that Hg was a major contributor to high pollution index values among a variety of heavy metals.

#### 3.4.2 Contamination degree (C_d_)

The degree of water contamination is quantified by water contamination index (C_d_). Cfi and C_d_ levels for fourteen water samples were calculated using equations (6), (7)), and Fig. 4b and Table S11 demonstrate the findings. According to classified contamination factor i.e. Cfi <1 = low contamination, 1≤ Cfi ≤3 = moderate contamination, 3≤ Cfi ≤6 = considerable contamination, Cfi ≥6 = very high contamination as described by Hakanson [54], indicates Hg has the highest level of contamination and other metals have low level of contamination. In the study the contamination factor was found in the order of Hg > Fe > Mn > Cu > Pb > Se > As > Cd > Zn > Cr > Ni in the studied river water (Table S11). The experiment's C_d_ levels were categorized as very high (C_d_ ≥ 24), considerable (12≤C_d_ < 24), moderate (6≤C_d_< 12), and low (C_d_ < 6) (Table 1). This investigation shows that contamination index values vary with locations, ranging from 1.59 to 22.98, with an average of 8.76 (Table S11), indicates low to high contamination degree depending on different locations. Considering the mean value the Hg has moderate contamination degree and other metals have low degree of contamination. The computed contamination degree of heavy metals in sampling points found in the order of S6>S13>S14>S12>S7>S4>S2>S5>S1>S3>S9>S8>S10>S11. Among locations, S8, S9, S10, S11, S3 have low contamination degree; S1, S2, S4, S5, S7 have moderate degree of pollution and S12, S13, S14, S6 have high contamination degree. Another study reported moderate to very high contamination of Ganga River [117].

**Table 1:** The degree of pollution in water based on contamination degree, nemerow pollution index and ecological risk index, risk index and water quality index.

Item	Pollution degree	References
P_N_ ≤ 1	Clean	[53]
1<P_N_ ≤ 2.5	Low	
2.5<P_N_ ≤ 7	Moderate	
P_N_ > 7	High	
$E_{r}^{i}\leq 40$	Low	[53,54,176]
${40<E}_{r}^{i}\leq 80$	Moderate	
${80<E}_{r}^{i}\leq 160$	Considerable	
${160<E}_{r}^{i}\leq 320$	High risk	
$E_{r}^{i}>320$	Very high risk	
C_d_ < 6	Low	[47,52,55]
6≤C_d_ < 12	Moderate	
12≤C_d_ < 24	Considerable	
C_d_ ≥ 24	Very high	
RI < 110	Low risk	[[53], [54], [55],^178^]
110≤RI < 200	Moderate	
200≤RI < 400	Considerable	
RI ≥ 400	Severe	
WQI = 0-25	Excellent	[179,180]
WQI = 26-50	Good	
WQI = 51-75	Poor	
WQI = 76-100	Very poor

**Table 2:** Comparison of mean concentration of heavy metals in water of Turag River with different other studies in the world.

Study area	Unit	Pb	Cd	Cr	Ni	Fe	Mn	Zn	Cu	As	Se	Hg	References
Buriganga river	μg/L	65.5	9.34	587.20									[19]
Shitalakhya river	mg/L	0.016	0.003	0.018	0.014		0.179	0.056	0.022	0.01			[84]
Buriganga river	mg/L	0.0655	0.0093	0.587	0.0088	–	–	–	0.163	–	–	–	[94]
Karatoa river	mg/L	–	–	0.005	0.005	–	0.101	–	–	–	–	–	[162]
Khiru river, Mymensingh	mg/L	0.0107	0.178	–	–	–	0.095	0.006	0.0037	–	–	–	[163]
Shitalakhya river	mg/L	0.005	0.003	0.005	0.005	–	–	0.106	–	–	–	–	[164]
Balu river (winter season)	mg/L	–	1.02	–	–	0.16	–	0.38	0.16	–	–	–	[165]
Balu River (Rainy season)	mg/L	–	–	–	–	0.04	0.05	0.08	–	–	–	–	[165]
Paira river (winter)	mg/L	0.0288	0.0009	0.057	0.0406	–	–	–	0.0351	–	–	–	[93]
Paira river (summer)	mg/L	0.0214	0.0005	0.033	0.0279	–	–	–	–	–	–	–	[93]
Korotoa river, Bogra (Summer)	mg/L	0.027	0.008	0.073	0.032	–	–	–	0.061	–	–	–	[93]
Korotoa river, Bogra (Winter)	mg/L	0.035	0.011	0.083	0.039	–	–	–	0.073	–	–	–	[93]
Buriganga river, Dhaka	mg/L	0.119	–	0.114	0.15	0.612	0.157	0.332	0.239	–	–	–	[166]
Karnaphuli river	mg/L	0.0168	–	0.086	–	–	–	–	–	–	–	–	[167]
Mayur river, Khulna city	mg/L	0.0045	–	–	–	0.15	–	–	1.33	0.003	–	–	[168]
Barani river area, Rajshahi	mg/L	0.008	0.90	–	–	0.50	–	–	2.67	0.04	–	–	[168]
Rupsha river	mg/L	0.0071	0.001	0.007	0.0039	–	–	–	0.00536	0.0054	–	–	[34]
Meghna river basin	mg/L	0.0044	0.003	0.014	0.005	1.139	0.018	0.036	–	0.011	–	–	[169]
Kortoya river basin	mg/L	0.0044	0.011	0.006	0.007	0.44	0.16	0.051	–	0.0018	–	–	[169]
Shitalakhya river basin	mg/L	0.015	0.0018	0.056	0.024	1.002	0.192	0.083	–	0.0083	–	–	[169]
Teesta river basin	mg/L	0.0048	0.0046	0.003	0.004	9.23	0.67	0.046	–	0.0017	–	–	[169]
Pasur river basin	mg/L	0.026	0.0098	0.04	0.021	2.062	0.065	0.033	–	0.012	–	–	[169]
Rupsha river basin	mg/L	0.0184	0.0065	0.0452	0.0187	0.9766	0.2885	0.0684		0.00352			[169]
Buriganga river	mg/L	BDL	BDL	0.002	0.002	0.006	–	0.01	0.005	0.0035	–	–	[170]
Bhairab river	μg/L	23.82	1.44	31.74	–	–	–	–	–	3.55	–	–	[126]
Halda river	μg/L	1283.3	12.5	70.8		402.5	469.5	549.4	85.85	60			[91]
Gomti river	mg/L	0.053	0.006	–	–	–	0.03	–	–	0.022	–	0.122	[142]
Southeastern Black sea	mg/kg	93.71	0.99	120.75	44.93	56,659.83	1168.53	155.03	82.66	13.66		0.18	[181]
Hoogly river, India	mg/kg	10.7	0.16	31.8	19.8	–	–	–	–	4	–	0.02	[182]
Yangtze river estuary, china	mg/kg	25.8	0.13	34.4	–	–	–	–	–	–	–	–	[183]
Southeast Black Sea (sediment)	mg/kg	41.37	0.20	60.46	27.29	27,646.37	571.87	94.16	45.66	7.36	–	–	[184]
Turnasuyu Stream, Ordu, Turkey (sediment)	mg/kg	60.23± 16.03	0.27 ± 0.13	5.67 ± 1.07	4.35 ± 1.5	15,080 ± 2194	361 ± 75	33.06 ± 5.69	19.1 ± 3.67	0.193± 0.246	–	–	[185]
Turag river (mean value ± SD)	μg/L	0.61 ± 0.72	0.13 ± 0.09	0.67 ± 0.85	1.20 ± 0.38	244.72 ± 214.35	28.93 ± 29.64	22.97 ± 10.93	8.28 ± 5.99	1.34 ± 0.39	–	8.23 ± 6.58	Our study
Standard limit	μg/L	50 [69,82]	10 [69,82]	100 [70]	200 [85]	200 [85]	1000 [86]	200 [85]	2000 [85]	100 [86]	20 [69,82]	1 [69,82]	[69,70,82,85,86]

#### 3.4.3 Nemerow pollution index (P_N_)

Nemerow pollution index (P_N_) was used to measure pollution levels at the specified sampling sites (Fig. 4c). This study used equation (8) to calculate the P_N_ index using Pb, Cd, Cr, Ni, Fe, Cu, Mn, Zn, As, Se, and Hg (n = 11). The level of pollution was categorized using Chen's classification system (53) (Table S7). The P_N_ index was highest at sample location S4 and lowest at sampling location S6 (Fig. 4c). P_N_ values varies from 58 to 665 among the sampling locations. Based on the P_N_, the pollution level at every sampling site is categorized as high and raising concern.

The effect of metal contribution in water can be estimated from different pollution indicators such as heavy metal pollution index (HPI), contamination degree (C_d_), nemerow pollution index (P_N_). The contamination degree (C_d_) is a versatile tool to identify the contamination level in environmental matrix. Contamination degree accounts for the degree of water pollution within a river due to the presence of contaminants like heavy metals as a whole. Heavy metal pollution index (HPI) is a potential tool applied to evaluate the composite influence of heavy metals in water. Nemerow pollution index (P_N_) is more comprehensive tool to find out the most pollution factor taking into consideration of other factors that reflects the water quality of each monitoring station.

#### 3.4.4 Ecological risk index (ERI)

The ERI evaluated heavy metal contamination risks to ecosystems and humans. Pb, Cd, Cr, Zn, Cu, As, and Hg were used to calculate the ERI at fourteen sampling sites using equations (9), (10)). Results for potential ecological risk factors ($E_{r}^{i}$) and risk index (RI) are shown in Fig. 4d and (e) and summarized in Table S5. The ecological risk hierarchy was found in the following order of Hg > Cd > As > Cu > Zn > Pb > Cr. Hg was above 320 in all points with an average of 1316.531, indicating significant ecological risk (Table 1). Pb, Cd, Cr, Cu, Zn, and As were below 40 (Table S5), indicating low ecological risk [54,118]. Considering ecological risk factor, Turag River water risk index (RI) at different sample points was S6>S13>S14> S12>S7>S2>S5>S4>S1>S3>S9>S8>S10>S11 (Fig. 4e and Table S5). S11 was found at moderate risk, whereas S10 and S8 were at considerable risk, with values exceeding 110 and 200, respectively (Table 1) [54,119]. All other locations were at severe risk, with a mean value of 1322.32 (Table S5). Ecological risks and potential ecological risks were found in the high-risk category at Kannauj, Rajghat, and Howrah sites of the Ganga River [118].

#### 3.4.5 Water quality index (WQI)

**Fig. 5:**
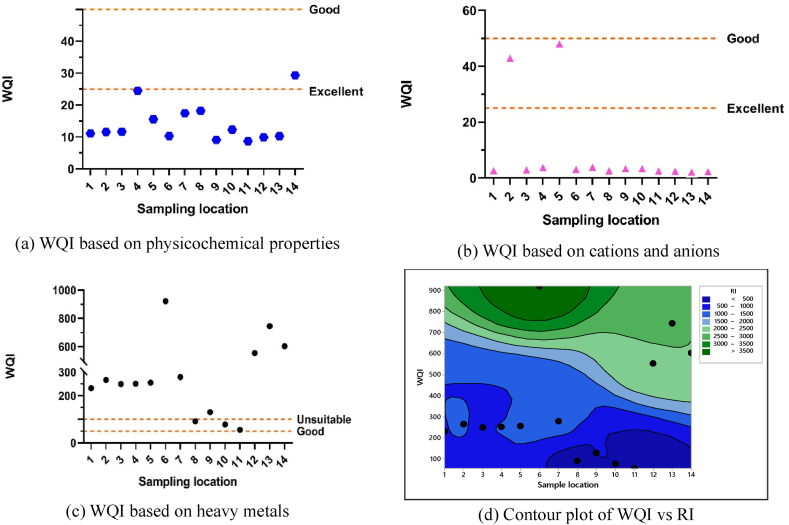
The water quality index (WQI) based on: (a) physicochemical properties, (b) cations and anions (c) heavy metals of Turag River at different locations where red dot lines represent the threshold value and (d) contour plot considering WQI and RI of river water. (For interpretation of the references to color in this figure legend, the reader is referred to the Web version of this article.)

The WQI facilitates water management [56,119,120]. This study usually uses the WQI to compress data on multiple parameters into one simple value [121,122]. This study calculated the WQI using 25 parameters (n = 25) (Table S6), including pH, EC, TDS, DO, COD TH, SAR, MAR, and other chemical parameters like F^−^, Cl^−^, NO_3_^−^, SO_4_^2−^, Ca, Mg, Na, K, and trace metals like Pb, Cd, Cr, Ni, Fe, Mn, Zn, Cu, As, Se, and Hg using the equations of 11–14. WQI values were good to excellent for physicochemical parameters as well as cations and anions. Locations S-13, S-6, S-12, and S-14 have higher WQI values for heavy metals. WQI ranged from 55 to 921, with a mean of 337.05, indicating poor water quality (Fig. 5 and Table S6).

The contour plot (Fig. 5d) depicts the Turag River WQI and RI distribution. WQI is on the Y-axis, while RI is in the colors. Tables S5 and S6 indicated sample point S6 had the highest WQI and RI. The lowest WQI and RI were 55.28 and 223.19 at sampling location S11. The finding after analyses of various indices indicates that Turag River water is polluted with heavy metals as they crossed the standard limit of the HPI score. The contamination degree (C_d_) has been computed to be moderate to high, while the pollution level is classified as high according to the guidelines of P_N_. The ecological risk is of great concern as there is an excessive presence of Hg, resulting in a severe overall risk index for the river water. So, the overall water quality index indicates that river water is unsuitable in terms of heavy metals for multipurpose like fishing, household activities, consumption, and irrigation purposes.

#### 3.4.6 Significance of environmental indices on water quality

Evaluation of the impact of metals on water quality can be done using different pollution indicators, such as heavy Metal Pollution Index (HPI), contamination degree (C_d_), nemerow pollution index (P_N_), ecological risk index (ERI), and water quality index (WQI). The contamination degree (C_d_) is a highly effective tool for assessing pollution and contamination levels in various environmental matrices. C_d_ accounts for the degree of water pollution as well as provides a straightforward method for identifying pollution levels within a river due to the presence of contaminants like heavy metals as a whole. But sometimes it may oversimplify complex pollution scenarios and lacks specificity regarding the sources or types of contaminants. So, the other pollution indices were conducted for a better understanding of the pollution and contamination status of the river water. The heavy metal pollution index (HPI) provides a valuable tool for assessing the overall influence of heavy metals on water quality. The HPI offers a quantitative assessment of heavy metal contamination, aiding in comparative analyses across locations and timeframes. But the HPI relies on predetermined weighting factors that may not accurately reflect local conditions or variations in metal toxicity. To overcome the limitations of HPI, the nemerow pollution index (P_N_) was carried out to understand the overall quality of river water. Nemerow pollution index (P_N_) is more comprehensive tool to find out the most pollution factor taking into consideration of other factors that reflects the water quality of each monitoring station. In addition, the ecological risk index (ERI) provides a comprehensive assessment of ecological risk, incorporating multiple parameters to evaluate the health and integrity of ecosystems. ERI serves as an early warning system for identifying emerging ecological threats, allowing for timely intervention and mitigation measures. WQI is a method that simplifies water quality data by converting it into a single numerical value. It takes into account various physicochemical and biological parameters, offering a thorough assessment of water quality and ecosystem health. The WQI has the potential to oversimplify the intricate dynamics of water quality, which could result in the oversight of subtle differences and localized variations in pollutant sources and ecological impacts. The outcomes of the WQI have the potential to increase public awareness regarding water quality concerns. This, in turn, can foster community involvement and garner support for conservation initiatives.

### 3.5 Human health risk assessment

The presence of chemical substances in river water poses two types of health risks: carcinogenic and non-carcinogenic. The mentioned risks can have negative impacts on human health.

#### 3.5.1 Carcinogenic risk

**Fig. 6:**
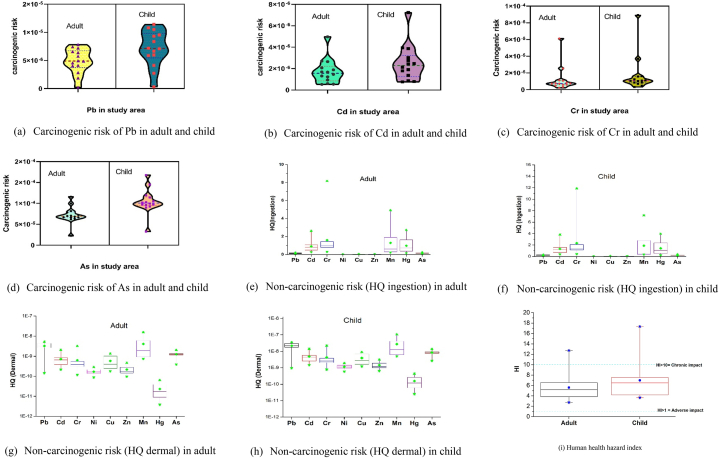
Carcinogenic risk and non-carcinogenic risk of different metals (a, b, c, d, e, f, g & h) along with human health hazard index (i) in adult and child.

Long-term exposure to some compounds can cause cancer. IARC designated Cr, Ni, Cd, Pb, and As as carcinogenic [[123], [124], [125], [126], [127]]. Pb, Cd, Cr, and As carcinogenicity for adults and children was assessed using equations (20), (21)). Fig. 6 (a, b, c, d) demonstrated that the results varied from 10^−4^ to 10^−6^ and depicts the spatial distribution of metals based on their median values at different locations. As had the highest TCR values, 6.9x10^−5^ for adults and 1x10^−4^ for children. The carcinogenic risk levels were ranked as As > Cd > Pb > Cr for adults and As > Pb > Cr > Cd for children. All metals TCR values, except for As, were below the level of 1 × 10^−6^ to 1x10^−4^ set by the US Environmental Protection Agency (71). The median As value for children was 1x10^−4^, close to the limit (Fig. 6d). Pb and Cr in children may cause carcinogenic consequences over time, as shown by the median values' near to the threshold values (Pb = 1.09x10^−5^ and Cr = 1.06x10^−5^), therefore contamination levels must be controlled (Fig. 6 a,c). Cr, Ni, Cd, Pb, and As had carcinogenic risk levels of 1x10^−6^ to 1x10^−4^ for all exposure pathways in adults and children except ingestion. TCR value for the same metals in the Buriganga River was observed below 1x10^−5^ [125,128]. Similar results was found in the Bhairab, Rupsha, and Yangtze rivers [[129], [130], [131]]. Cr, Cd, and As damage DNA, create genomic instability, and inhibit DNA repair processes after oxidative stress [132]. Chronic water exposure, especially in children, can cause cancer, hypertension, and neuropathy [133].

#### 3.5.2 Non-carcinogenic risk

Assessing non-carcinogenic risks is crucial for human and environmental health. The Hazard Quotient (HQ) and other approaches estimate non-carcinogenic risks using equations (15), (16), (17), (18), (19)). Comparing pollutant exposure to safe reference doses or concentrations identifies health risks and prioritizes pollutant control. Fig. 6 (e-h,) demonstrates non-carcinogenic risk in adult and children. Heavy metals (Pb, Cd, Cr, Ni, Cu, Zn, Mn, Hg, As) in Turag River water samples pose non-carcinogenic risks for adults and children (Fig. 6 e-h). Considering average values, adult and child pathway ingestion hazard quotients (HQ) were in the order of Mn > Cr > Hg > Cd > As > Pb > Cu > Zn > Ni and Cr > Mn > Hg > Cd > As > Pb > Cu > Zn > Ni. The ingestion of river water at the study sites was the primary route for the accumulation of Hg, Mn, Cr, and Cd in the bodies of adults and children. These components' average concentrations exceed the USEPA standard of 1 [69]. HI and HQ levels >1 indicate high health risks due to long-term contact with contaminated water [61,67,131]. In Fig. 6 (e, f, g, h), the mean values for Hg, Mn, Cr, and Cd were 0.98, 1.29, 1.88, and 0.43, respectively. Dermal contact for both demographic groups lowered the HQ below 1. At all locations, the HI surpassed threshold value 1 [131,134], averaging 5.61 and 6.99 (Fig. 6i) for adults and children (Table S8). Metals in water and skin contact are more likely to affect both demographic groups, especially children, chronically. Pb and As pose substantial health risks to adults and children [128]. The Bhairab, Rupsha, and Yangtze Rivers had high non-carcinogenic risk [[128], [129], [130]]. The presence of hazardous materials can disrupt various biological processes, including growth, reproduction, differentiation, damage repair, and death [132].

### 3.6 Correlation matrix

The Pearson correlation matrix of heavy metals is presented in Table 3. Statistical significance is indicated by p-values below 0.05. Findings show that the Cr–Ni, Cr–Zn and Ni–Zn pairs have a positive correlation (0.584, 0.603, 0.645) at the significance level of p < 0.05 which suggests that Cr, Ni, and Zn may share a common source of origin. Moreover, a strong positive relationship of both Zn–Cu (0.731) and Cu–Se (0.731) pairs was observed at the significance level of p < 0.01 that reveals Cu, Se, Zn may have same origin. However, As has positive correlation (0.535) with Zn at significance level of p < 0.05 and negative significant correlation (−0.660) with Fe at the level of p < 0.05. Significant positive correlations between the heavy metals imply that they might originate from the same source, whereas negative correlations suggest that they might originate from separate sources.

**Table 3:** The Pearson correlation matrix of heavy metals.

Correlations											
	Pb	Cd	Cr	Ni	Fe	Mn	Cu	Zn	As	Se	Hg
Pb	1										
									
Cd	0.343	1									
	0.230										
Cr	0.102	−0.125	1								
	0.729	0.669									
Ni	−0.070	−0.276	0**.584**^a^	1							
	0.813	0.339	0.**028**								
Fe	−0.482	−0.381	−0.019	0.470	1						
	0.081	0.179	0.950	0.090							
Mn	0.029	−0.332	0.154	0.144	0.145	1					
	0.923	0.246	0.599	0.622	0.620						
Cu	−0.277	−0.130	0.523	0**.641**^a^	0.327	0.148	1				
	0.337	0.658	0.055	0**.013**	0.253	0.614					
Zn	0.268	0.025	0**.603**^a^	0**.635**^a^	−0.011	0.159	0.**731**^b^	1			
	0.355	0.933	0**.022**	0**.015**	0.971	0.586	0**.003**				
As	0.415	0.117	0.376	0.274	**−**0**.660**^a^	0.073	0.119	0**.535**^a^	1		
	0.140	0.691	0.185	0.342	0**.010**	0.804	0.686	0**.048**			
Se	−0.342	0.116	0.051	0.169	0.094	0.160	0**.731**^b^	0.522	0.082	1	
	0.232	0.692	0.862	0.564	0.748	0.584	0**.003**	0.056	0.781		
Hg	0.435	0.391	−0.108	−0.099	−0.177	−0.423	−0.259	−0.266	−0.032	−0.328	1
	0.120	0.167	0.713	0.736	0.544	0.132	0.372	0.359	0.915	0.252	

Cell Contents: Pearson correlation.

P-Value.

a . Correlation is significant at the 0.05 level (2-tailed).

b . Correlation is significant at the 0.01 level (2-tailed).

**Table 4:** Concentrations of certified reference material (NRC-CNRC, SLRS-6: River Water Certified Reference Material for Trace Metals and other Constituents), measured concentrations, and recoveries for metals.

Metal	Certified value (μg/L)	Detected value (μg/L)	Recovery (%)
Pb	0.170 ± 0.026	0.1615 ± 0.025	95.0
Cd	0.0063 ± 0.0014	0.00663 ± 0.0015	105.2
Cr	0.252 ± 0.012	0.244 ± 0.014	96.8
Ni	0.616 ± 0.022	0.609 ± 0.015	98.9
Fe	84.3 ± 3.6	83.61 ± 1.9	99.2
Mn	2.12 ± 0.10	2.08 ± 0.09	98.1
Cu	23.9 ± 1.8	24.37 ± 1.2	102.0
Zn	1.76 ± 0.12	1.77 ± 0.06	100.6
As	0.57 ± 0.08	0.578 ± 0.03	101.4
Se	–	–	–
Hg	–	–	–

### 3.7 Principal component analysis (PCA)

**Fig. 7:**
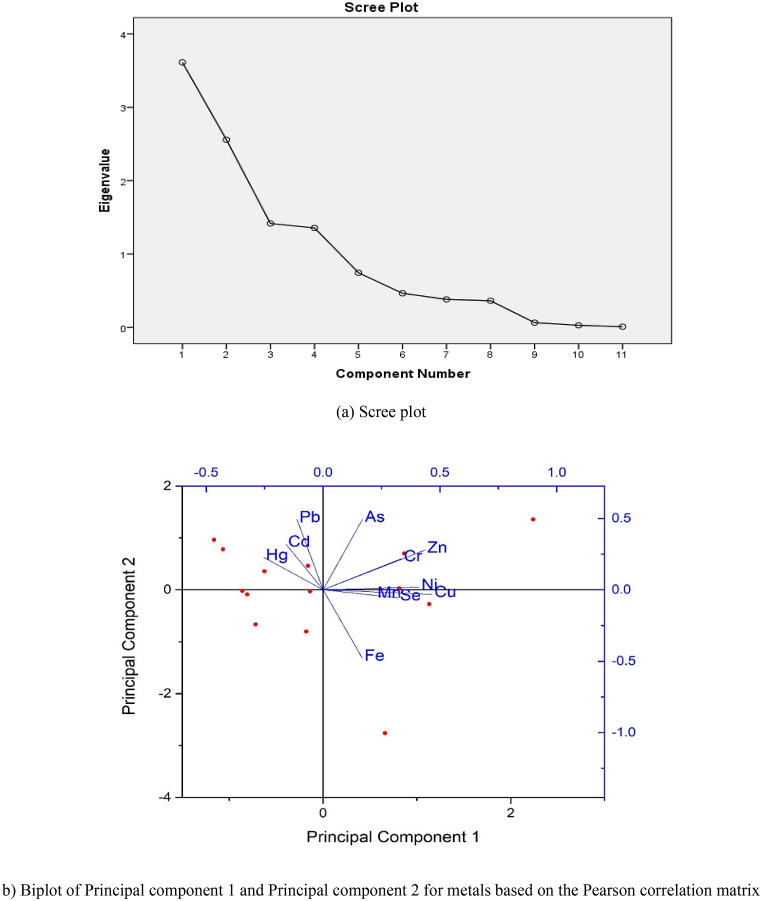
(a) Scree plot of the characteristic roots and (b) Biplot of Principal component 1 and Principal component 2 for metals based on the Pearson correlation matrix component plot of principal component analysis.

The principal component (PC) analysis was carried out by applying varimax rotation on the dataset of the metals to identify the contributing factors and possible sources of the pollutants in water of the Turag River (Tables S9 and S10). Four PCs with Eigenvalues greater than 1 were identified from principal component analysis (PCA) applied to the standardized dataset of heavy metals. Fig. 7 (a) shows the scree plot of eigenvectors and principal component number, and Fig. 7 (b) shows the Biplot of Principal component 1 and Principal component 2 for metals based on the Pearson correlation matrix. Based on Kaiser criterion the selection of dominant principal components was determined which states components with eigenvalues greater than 1 should be considered [132]. PC1 has an eigenvalue of 3.61 and explains 32.84 % of the data variance and is highly loaded with Cu (0.886), Zn (0.829), Ni (0.781), and Cr (0.638) and Se (0.606). The primary sources of Cu in the environment are agricultural fertilizers and pesticides, agrochemical enterprise, mining operations, chemical, pharmaceutical, and paper manufacturing industries [135]. Beside Se is released from anthropogenic sources like metallurgical, glass, and pigment-producing industries as well as from geological sources [136,137]. Zn may be released from fuel oil burning and untreated dying industrial wastes [[138], [139], [140]]. Cr was found in the study area due to the direct disposal of untreated wastes from leather tanning, dyes and colorants, chrome plating, commercial welding, and timber preservation upstream agricultural waste runoff, paint and inks, textile chemicals, and so on [141,142]. Ni is transported into aquatic environments by anthropogenic activities, including industrial operations such as power generation, steel manufacturing, and incineration of waste products [143]. PC2 has an eigenvalue of 2.56, accounted for 23.25 % of the data variance and has high positive loadings of As (0.789), Pb (0.787), Cd (0.512). Arsenic mostly comes from geological sources, which release the element through various weathering processes [[144], [145], [146]] and mining, industrial activities, metal processing, fertilizers, and pesticides are the major sources of As intrusion in this aquatic environment may be [147,148]. The steel industry, runoff from boats and steamers, batteries, lead-based paints or pigments, corroded lead pipes in soft water areas, sewage sludge, Pb-containing glass, and oil leaks are some of the major sources of lead pollution in water [[149], [150], [151]]. the primary sources of Cd in the study area are the metal working industry, the use of chemical fertilizers and pesticides, the production and processing of plastics, and the disposal of trash from chemical plants and battery industries [[152], [153], [154], [155], [156]]. PC3 has eigenvalue 1.42, and it can explain 12.87 % of the data variance with higher loading of Hg (0.525) and Cd (0.507) and PC4 has eigenvalue 1.36 and 12.33 % of data variance with positive loadings of Ni (0.483), Cr (0.391), Fe (0.339), Hg (0.322). River water can get Hg from a variety of sources, including weathering and industrial processes, burning coal, and the use of fertilizers and pesticides that contain mercury [157]. Various sources of Fe release into rivers include natural processes like weathering and erosion as well as human-caused ones such sewage systems and residential effluents [158]. Along with geological sources, industrial activities, coal burning, use of mercury-containing pesticides and fertilizers are the contributing sources for Hg intrusion in water body [157]. Fe can be released both naturally through weathering and erosion of rocks and minerals and in anthropogenic way by discharging effluents and waste water from household activities and sewage system into river [158]. The findings revealed the association among the metals and identified that the origin of metals from untreated industrial effluent, urban wastes and other geological sources [159]. Therefore, along with natural sources industrial and agricultural chemicals as well as anthropogenic activities are the contributory sources for the heavy metals in water [160,161].

### 3.8 Hierarchical cluster analysis (HCA)

**Fig. 8:**
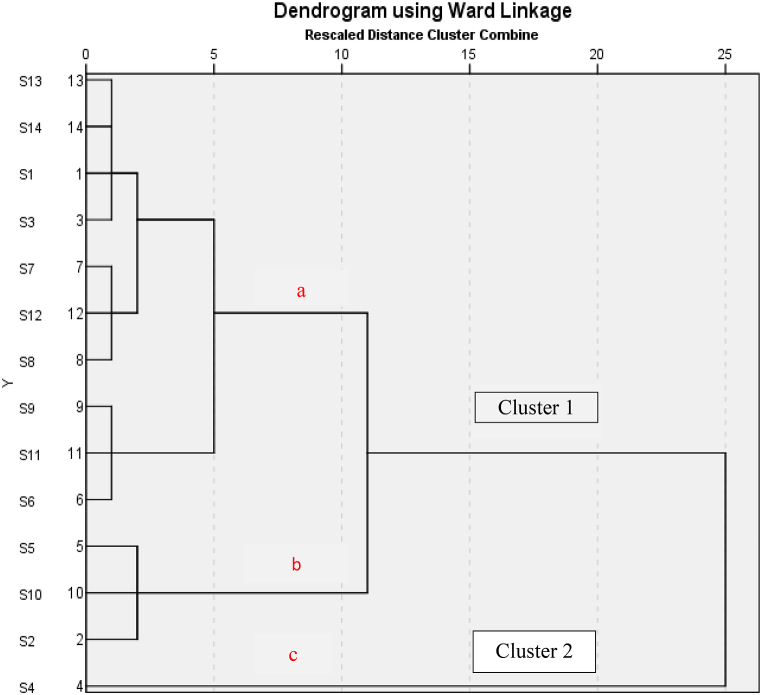
Multivariate hierarchical cluster analysis among studied locations by ward linkage.

Similarity or dissimilarity among studied locations was investigated through multivariate hierarchical cluster analysis (HCA) is illustrated in Fig. 8 [171]. The analysis yielded two clusters that were found to be statistically significant and distinct. The first cluster was further divided into two sub-clusters, denoted as (a) and (b). Sub-cluster (a) included stations S1, S3, S6, S7, S8, S9, S11, S12, S13, and S14, while sub-cluster (b) consisted of stations S5, S10, and S2. The second cluster, referred to as subclass (c), consisted solely of station S4. These clusters were identified based on similarities in the sources of contamination, as shown in Fig. 8. The significant pollution load stemming from industrial operations and human activity could potentially be attributed to the distinct clustering of these stations in the analysis.

The pollution stress caused by various industries, including paper cement, chemical, fertilizer, pharmaceutical, dyeing, and textile industries, as well as nearby power stations, is responsible for the environmental degradation in these areas [168,172]. Cluster 1 and cluster 2 may have variations in the sources of pollution [173]. Within the subclasses, one category encompasses stations located in close proximity to anthropogenic sources such as aquaculture, agricultural activities, paper industry, dyeing industry, textile industry, and shipping activity. Another category includes stations near the chemical industry and urban waste. The third category consists of stations located at the discharge point of industrial effluents [174,175].

**Fig. 9:**
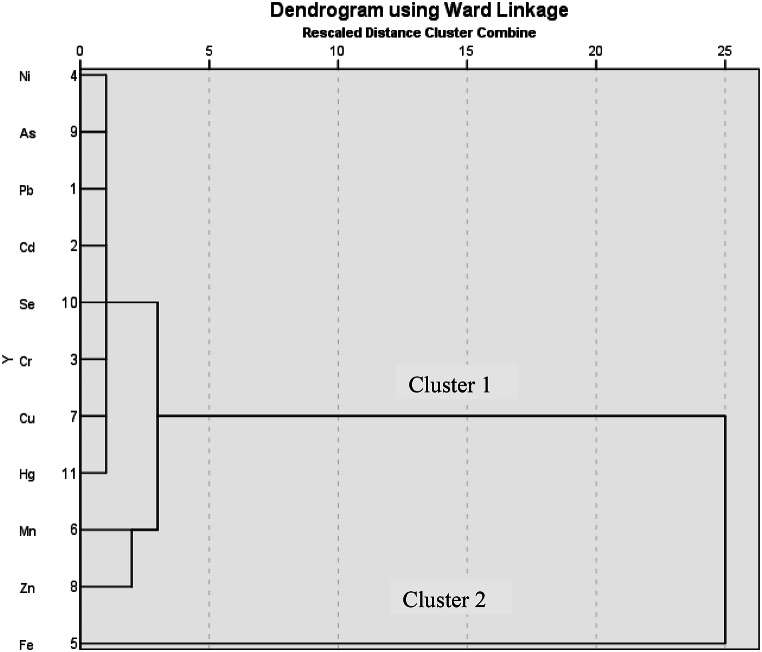
A hierarchical cluster analysis among metals following Ward linkage method.

The cluster analysis was also employed to explore the relationship between the factors and their probable contamination sources [176,177]. HCA with ward linkage method is further employed to explore the associations between heavy metals (Fig. 9). Two clusters are distinguished: the first cluster subdivision is primarily composed of Ni, As, Pb, Cd, Se, Cr, Cu, Hg along with Mn and Zn and the second cluster is composed of Fe solely. This analysis reveals that comparing the metals, cluster 1 would be more harmful than cluster 2. Probably Zn, Mn are associated with each other by same source of origin. It also indicates that the major sources of Fe into river water are different from others.

## 4 Conclusion

The findings of this study indicate that the water quality meets basic standards across various measures. The pH, EC, TDS, DO, COD, TH, SAR, MAR, Na, K, Ca, Mg, Cl^−^, F^−^, NO_3_^−^, and SO_4_^2−^ values were found within the acceptable range. The heavy metal pollution index and Nemerow multi-factor index indicates severe pollution levels. Degree of water contamination ranges from low-moderate-significant, according to the contamination index. A complete examination of the carcinogenic risk of Pb, Cd, Cr, and As in adults and children was computed and found that As is the most carcinogenic constituent for both population groups, particularly for children is concerning. The hazard quotient of individual pathway ingestions can be ordered in a strict manner as follows: Mn > Cr > Hg > Cd > As > Pb > Cu > Zn > Ni for adults, and Cr > Mn > Hg > Cd > As > Pb > Cu > Zn > Ni for child. The human health risk index exceeds 1 in all assessed locations. This alarming discovery confirmed a major risk to both adults and children. Although the principal component analysis strongly suggests that effluent discharge from the surrounding industries is the primary source of heavy metal contamination in the water of the Turag River. In the end, The principal component analysis indicated that the main source of heavy metal contamination in the Turag river water is due to the direct discharge of effluents from nearby industries and the runoff of agrochemicals from nearby agricultural land. In the studied areas, the outlets were discovered within a shared drainage system utilized by a cluster of industries. This shared system made it difficult to precisely attribute specific types of effluents to their respective industrial sources. Consequently, the accurate identification and characterization of effluents based on their originating industries were challenging. Parameters for human exposure and trace metal toxicity were mainly based on USEPA guidelines and international research to reduce inaccuracies. Despite these precautions, inherent uncertainties from individual exposure variability, population susceptibility differences, and gaps in toxicological data remain. These uncertainties may affect the reliability of health risk assessments, highlighting the need for ongoing research and refined methodologies. Expanding sampling sites across the Turag River and incorporating localized data and individual-specific factors will enhance accuracy. Continuous research and methodological advancements are essential to further reduce uncertainties and improve risk assessment reliability. Based on the study's findings, authorities must act immediately to prevent the discharge of untreated industrial effluents from nearby industries. Failure to do so might adversely affect the environment and public health. Thus, this issue requires immediate and strict action. To preserve the water quality of the Turag River and protect its invaluable economic value, immediate action to prevent any further deterioration of its water quality must be taken.

## Funding sources

The research endeavors detailed herein were made possible through the generous financial support provided by the 10.13039/501100005999Bangladesh Council of Scientific and Industrial Research (BCSIR), situated in Dhaka-1205, Bangladesh. This critical contribution of financial resources significantly facilitated the execution of regular research and development (R&D) activities. Furthermore, this research did not receive any specific grant from funding agencies in the public, commercial, or not-for-profit sectors.

## CRediT authorship contribution statement

**Priyanka Dey Suchi:** Writing – original draft, Software, Methodology, Investigation, Formal analysis, Data curation, Conceptualization. **Md Aftab Ali Shaikh:** Writing – review & editing, Supervision, Resources. **Badhan Saha:** Writing – review & editing, Software, Project administration, Methodology, Investigation, Formal analysis, Data curation, Conceptualization. **Mohammad Moniruzzaman:** Resources, Formal analysis. **Md Kamal Hossain:** Formal analysis. **Afroza Parvin:** Formal analysis. **Afsana Parvin:** Formal analysis.

## Declaration of competing interest

The authors declare that they have no known competing financial interests or personal relationships that could have appeared to influence the work reported in this paper.
