# Supplementary material for: Comprehensive index analysis approach for ecological and human health risk assessment of a tributary river in Bangladesh
Source: Heliyon. 2024 Jun 6;10(13):e32542. doi: 10.1016/j.heliyon.2024.e32542 (PMC11260973; doi:10.1016/j.heliyon.2024.e32542)
Supplement: Multimedia component 1 [file mmc1.docx]

**Comprehensive index analysis approach for ecological and human health risk assessment of a tributary river in Bangladesh**

Priyanka Dey Suchi^a^, Md. Aftab Ali Shaikh^a,b^, Badhan Saha^a*^, Mohammad Moniruzzaman^a^, Md. Kamal Hossain^a^, Afroza Parvin^a^, Afsana Parvin^a^

^a^ Bangladesh Council of Scientific and Industrial Research (BCSIR), Dhaka-1205, Bangladesh

^b^ Department of Chemistry, University of Dhaka, Dhaka-1000, Bangladesh

**Corresponding author: Badhan Saha**

**Senior Scientific Officer**

Bangladesh Council of Scientific and Industrial Research (BCSIR),

Dhaka-1205, Bangladesh

E-mail address: [badhanbcsir@gmail.com](mailto:badhanbcsir@gmail.com)

**Supplementary Materials**

**Table S1** Difference of pH, EC, TDS, DO, TH, SAR, MAR and COD at different locations of Turag River

| **Sampling**  **Location** | **pH** | **EC (µS/cm)** | **TDS (mg/L)** | **DO (mg/L)** | **COD (mg/L)** | **TH (mg/L)** | **SAR (meq/L)** | **MAR (%)** |
| --- | --- | --- | --- | --- | --- | --- | --- | --- |
| S1 | 7.33 | 189.8 | 34.9 | 3.45 | 12 | 793.9 | 0.55 | 22.61 |
| S2 | 7.01 | 194 | 97.1 | 2.28 | 35 | 958.15 | 0.60 | 28.26 |
| S3 | 7.14 | 1367 | 683 | 3.67 | 30 | 1454.35 | 0.38 | 38.18 |
| S4 | 10.94 | 2600 | 1300 | 3.73 | 81 | 1561.3 | 0.25 | 2.43 |
| S5 | 6.68 | 982 | 491 | 2.94 | 167 | 1032.15 | 0.38 | 26.33 |
| S6 | 7.15 | 171.9 | 86 | 3.79 | 14 | 1184.05 | 0.39 | 19.23 |
| S7 | 8.77 | 314 | 156.8 | 2.23 | 55 | 1095.35 | 0.32 | 23.69 |
| S8 | 8.97 | 345 | 172.1 | 1.66 | 38 | 1189.05 | 0.27 | 18.75 |
| S9 | 7.24 | 151 | 75.6 | 5.67 | 9 | 1201.75 | 0.24 | 27.92 |
| S10 | 7.07 | 381 | 190.6 | 1.65 | 44 | 1253.75 | 0.20 | 19.26 |
| S11 | 7.34 | 139.8 | 70.1 | 5.93 | 11 | 976.25 | 0.22 | 15.88 |
| S12 | 7.44 | 140.3 | 70.2 | 5.58 | 15 | 1164 | 0.22 | 28.72 |
| S13 | 7.41 | 141.1 | 70.7 | 4.79 | 14 | 1084.4 | 0.21 | 19.67 |
| S14 | 7.39 | 140.6 | 70.3 | 5.57 | 1 | 1452.35 | 0.19 | 40.52 |
| Mean | 7.71±1.13 | 518.39±701.56 | 254.89±353.24 | 3.78±1.52 | 37.57±43.13 | 1171.49±210.87 | 0.32±0.13 | 23.67±9.44 |
| Standard value | 8.5 [69,82] | 300 [69,82] | 1000 [69,82,86] | 6 [86] | 50 [69,82] | 300 [69,82] | 9 [69,82] | 50 [69,82] |

**Table S2** Distribution of anions in river water

| **Sampling Location** | **F (mg/L)** | **Cl (mg/L)** | **NO_3 (_mg/L)** | **SO_4_ (mg/L)** |
| --- | --- | --- | --- | --- |
| S1 | 0.1329 | 6.9973 | 0.2992 | 33.6007 |
| S2 | 2.3742 | 18.557 | 0.1458 | 9.3163 |
| S3 | 0.1247 | 28.481 | 0.2042 | 111.1795 |
| S4 | 0.1788 | 33.8644 | 0.3891 | 139.69 |
| S5 | 2.6237 | 216.2754 | 0.5128 | 229.9361 |
| S6 | 0.1622 | 4.711 | 0.7788 | 22.883 |
| S7 | 0.2008 | 17.0824 | 0.1432 | 36.695 |
| S8 | 0.1292 | 20.6111 | 1.2048 | 45.7359 |
| S9 | 0.1752 | 3.2492 | 2.5861 | 18.7089 |
| S10 | 0.165 | 29.0475 | 1.921 | 43.0329 |
| S11 | 0.1302 | 2.106 | 1.6819 | 17.8743 |
| S12 | 0.1244 | 2.0677 | 0.8221 | 17.8769 |
| S13 | 0.1104 | 2.1716 | 0.672 | 17.8769 |
| S14 | 0.1124 | 2.0748 | 0.7349 | 18.936 |
| Mean(±SD) | 0.48±0.86 | 27.66±55.49 | 0.86±0.74 | 54.52±63.14 |
| Standard value | 1.5 [69,82] | 250 [69,82] | 50 [69,82] | 250 [69,82] |

**Table S3** Distribution of cations in river water

| **Sampling Location** | **Na (mg/L)** | **K (mg/L)** | **Ca(mg/L)** | **Mg(mg/L)** |
| --- | --- | --- | --- | --- |
| S1 | 1.56 | 0.49 | 12.29 | 3.59 |
| S2 | 1.85 | 0.52 | 13.75 | 5.42 |
| S3 | 1.46 | 1.43 | 17.98 | 11.11 |
| S4 | 0.99 | 1.27 | 30.47 | 0.76 |
| S5 | 1.21 | 1.63 | 15.21 | 5.44 |
| S6 | 1.35 | 0.46 | 19.13 | 4.56 |
| S7 | 1.06 | 0.50 | 16.72 | 5.19 |
| S8 | 0.93 | 0.62 | 19.32 | 4.46 |
| S9 | 0.84 | 0.44 | 17.33 | 6.71 |
| S10 | 0.71 | 0.84 | 20.25 | 4.83 |
| S11 | 0.69 | 0.41 | 16.43 | 3.10 |
| S12 | 0.76 | 0.44 | 16.60 | 6.69 |
| S13 | 0.70 | 0.42 | 17.42 | 4.27 |
| S14 | 0.72 | 0.41 | 17.28 | 11.77 |
| Mean(±SD) | 1.06±0.36 | 0.70±0.42 | 17.87±4.20 | 5.56±2.90 |
| Standard value | 200 [92] | 12 [92] | 75 [69,82] | 50 [69,82] |

**Table S4** Comprehensive distribution of trace metal in water at different locations

| **Sampling Location** | **Pb (ppb)** | **Cd (ppb)** | **Cr (ppb)** | **Ni (ppb)** | **Cu (ppb)** | **Fe (ppb)** | **Mn (ppb)** | **Zn (ppb)** | **As (ppb)** | **Se (ppb)** | **Hg (ppb)** |
| --- | --- | --- | --- | --- | --- | --- | --- | --- | --- | --- | --- |
| S1 | 2.157 | 0.138 | 0.270 | 1.181 | 7.734 | 163.853 | 6.562 | 27.998 | 1.462 | 0.269 | 5.73 |
| S2 | 2.445 | 0.091 | 0.188 | 1.185 | 4.810 | 369.305 | 42.264 | 30.081 | 1.155 | 0.135 | 6.59 |
| S3 | 1.602 | 0.108 | 0.125 | 1.562 | 9.728 | 147.098 | 60.374 | 33.139 | 2.160 | 0.821 | 5.05 |
| S4 | 0.071 | 0.040 | 0.332 | 1.644 | 14.454 | 936.304 | 14.731 | 17.312 | 0.418 | 0.633 | 6.20 |
| S5 | 2.105 | 0.125 | 3.427 | 2.002 | 18.120 | 274.886 | 43.318 | 48.881 | 1.862 | 0.615 | 6.31 |
| S6 | 2.572 | 0.199 | 0.397 | 0.726 | 6.446 | 81.763 | 5.292 | 18.118 | 1.275 | 0.306 | 22.73 |
| S7 | 1.328 | 0.044 | 1.424 | 1.511 | 15.529 | 204.151 | 32.888 | 26.137 | 1.527 | 0.251 | 6.89 |
| S8 | 0.931 | 0.195 | 0.403 | 0.987 | 18.933 | 182.298 | 42.699 | 34.217 | 1.243 | 1.896 | 2.25 |
| S9 | 0.615 | 0.069 | 0.602 | 0.843 | 2.548 | 104.469 | 6.202 | 10.304 | 1.306 | 0.181 | 3.22 |
| S10 | 1.625 | 0.038 | 0.418 | 0.972 | 3.301 | 306.206 | 110.273 | 11.822 | 1.186 | 0.181 | 1.93 |
| S11 | 1.503 | 0.153 | 0.621 | 0.625 | 2.864 | 107.318 | 5.345 | 17.334 | 1.233 | 0.193 | 1.36 |
| S12 | 2.375 | 0.123 | 0.425 | 1.258 | 4.487 | 202.079 | 12.225 | 18.635 | 1.248 | 0.169 | 13.68 |
| S13 | 1.351 | 0.096 | 0.469 | 1.137 | 3.524 | 175.567 | 11.433 | 11.907 | 1.344 | 0.123 | 18.38 |
| S14 | 1.891 | 0.366 | 0.329 | 1.108 | 3.458 | 170.787 | 11.398 | 15.684 | 1.316 | 0.144 | 14.87 |
| mean | 0.61±0.72 | 0.13±0.09 | 0.67±0.85 | 1.20±0.38 | 8.28±5.99 | 244.72±214.35 | 28.93±29.64 | 22.97±10.93 | 1.34±0.39 | 0.42±0.48 | 8.23±6.58 |
| Standard limit | 50 [69,82] | 10 [69,82] | 100  [70] | 200 [85] | 200 [85] | 1000 [86] | 200 [85] | 2000 [85] | 100 [86] | 20 [69,82] | 1 [69,82] |

**Table S5** Potential Ecological Risk Factor and Potential Ecological Risk Index of HMs in Turag River water with index values

| $\boldsymbol{E}_{\boldsymbol{r}}^{\boldsymbol{i}}$ | | | | | | | | | **RI** | |
| --- | --- | --- | --- | --- | --- | --- | --- | --- | --- | --- |
| **Sampling Location** | **Hg** | **As** | **Zn** | **Cu** | **Cr** | **Cd** | **Pb** | $\sum_{\boldsymbol{i=0}}^{\boldsymbol{n}} \boldsymbol{E}_{\boldsymbol{r}}^{\boldsymbol{i}}$ | |  |
| S1 | 916.979 | 0.975 | 0.160 | 0.773 | 0.006 | 4.154 | 0.154 | 923.20 | |  |
| S2 | 1055.040 | 0.770 | 0.172 | 0.481 | 0.004 | 2.724 | 0.175 | 1059.37 | |  |
| S3 | 807.965 | 1.440 | 0.189 | 0.973 | 0.003 | 3.229 | 0.114 | 813.91 | |  |
| S4 | 991.521 | 0.279 | 0.099 | 1.445 | 0.007 | 1.194 | 0.005 | 994.55 | |  |
| S5 | 1009.847 | 1.241 | 0.279 | 1.812 | 0.076 | 3.737 | 0.150 | 1017.14 | |  |
| S6 | 3636.000 | 0.850 | 0.104 | 0.645 | 0.009 | 5.975 | 0.184 | 3643.77 | |  |
| S7 | 1102.874 | 1.018 | 0.149 | 1.553 | 0.032 | 1.315 | 0.095 | 1107.04 | |  |
| S8 | 360.318 | 0.829 | 0.196 | 1.893 | 0.009 | 5.854 | 0.067 | 369.17 | |  |
| S9 | 515.735 | 0.871 | 0.059 | 0.255 | 0.013 | 2.065 | 0.044 | 519.04 | |  |
| S10 | 309.240 | 0.790 | 0.068 | 0.330 | 0.009 | 1.150 | 0.116 | 311.70 | |  |
| S11 | 217.280 | 0.822 | 0.099 | 0.286 | 0.014 | 4.586 | 0.107 | 223.19 | |  |
| S12 | 2188.800 | 0.832 | 0.106 | 0.449 | 0.009 | 3.688 | 0.170 | 2194.05 | |  |
| S13 | 2940.640 | 0.896 | 0.068 | 0.352 | 0.010 | 2.889 | 0.097 | 2944.95 | |  |
| S14 | 2379.200 | 0.877 | 0.090 | 0.086 | 0.007 | 10.976 | 0.135 | 2391.37 | |  |
| Mean | 1316.531 | 0.892 | 0.131 | 0.810 | 0.015 | 3.824 | 0.115 | 1322.32 | |  |

**Table S6** WQI of Turag River

| **Sampling Location** | Physicochemical parameter | Ions | Heavy metals |
| --- | --- | --- | --- |
| S1 | 11.09 | 2.82 | 232.52 |
| S2 | 11.56 | 43.15 | 267.40 |
| S3 | 11.63 | 3.06 | 250.03 |
| S4 | 24.48 | 3.93 | 251.58 |
| S5 | 15.58 | 48.24 | 256.13 |
| S6 | 10.32 | 3.3 | 921.94 |
| S7 | 17.43 | 4 | 279.54 |
| S8 | 18.19 | 2.78 | 91.61 |
| S9 | 9.11 | 3.57 | 130.66 |
| S10 | 12.27 | 3.51 | 78.46 |
| S11 | 8.69 | 2.67 | 55.28 |
| S12 | 9.9 | 2.62 | 554.90 |
| S13 | 10.29 | 2.33 | 745.46 |
| S14 | 29.36 | 2.48 | 603.24 |
| Mean | 14.28 | 9.69 | 337.05 |

**Table S7** HPI, contamination index and Nemerow index values of heavy metals

| **Sampling Locations** | **HPI score** | **Nemerow Index (P_N_)** |
| --- | --- | --- |
| S1 | 475.34 | 116.70 |
| S2 | 546.88 | 262.79 |
| S3 | 419.06 | 105.37 |
| S4 | 513.98 | 665.13 |
| S5 | 523.67 | 196.08 |
| S6 | 1883.92 | 58.51 |
| S7 | 571.63 | 145.57 |
| S8 | 187.44 | 130.21 |
| S9 | 267.33 | 74.34 |
| S10 | 160.6 | 218.34 |
| S11 | 112.82 | 76.41 |
| S12 | 1134.15 | 143.84 |
| S13 | 1523.55 | 124.99 |
| S14 | 1232.95 | 121.60 |
| Mean | 682.38 | 174.28 |
| Standard Deviation | 525.68 | 146.66 |

**Table S8** Hazard index of studied heavy metals for ingestion and dermal contact by adult and child in Turag River.

| Sampling Locations | HI (Adult) | HI (Child) | Recommended HI [131] |
| --- | --- | --- | --- |
| S1 | 3.01 | 3.85 | HI≤1 obvious adverse impact  HI>1 most probable adverse impact  HI>10 high or chronic of acute impact |
| S2 | 4.15 | 5.66 |  |
| S3 | 4.81 | 6.60 |  |
| S4 | 2.59 | 3.64 |  |
| S5 | 12.23 | 17.32 |  |
| S6 | 5.72 | 7.57 |  |
| S7 | 6.36 | 9.06 |  |
| S8 | 4.84 | 6.37 |  |
| S9 | 2.81 | 3.85 |  |
| S10 | 6.72 | 9.60 |  |
| S11 | 3.27 | 4.20 |  |
| S12 | 4.45 | 5.98 |  |
| S13 | 4.80 | 6.63 |  |
| S14 | 6.02 | 7.50 |  |
| Mean | 5.13 | 6.99 |  |

**Table S9** The factor loadings, cumulative percentage, and percentage of variance

| **Total Variance Explained** | | | | | | | | | |
| --- | --- | --- | --- | --- | --- | --- | --- | --- | --- |
| Component | Initial Eigenvalues | | | Extraction Sums of Squared Loadings | | | Rotation Sums of Squared Loadings | | |
|  | Total | % of Variance | Cumulative % | Total | % of Variance | Cumulative % | Total | % of Variance | Cumulative % |
| 1 | 3.612 | 32.838 | 32.838 | 3.612 | 32.838 | 32.838 | 2.805 | 25.503 | 25.503 |
| 2 | 2.558 | 23.254 | 56.092 | 2.558 | 23.254 | 56.092 | 2.386 | 21.687 | 47.190 |
| 3 | 1.416 | 12.870 | 68.962 | 1.416 | 12.870 | 68.962 | 1.898 | 17.256 | 64.446 |
| 4 | 1.356 | 12.327 | 81.289 | 1.356 | 12.327 | 81.289 | 1.853 | 16.842 | 81.289 |
| 5 | .745 | 6.771 | 88.060 |  |  |  |  |  |  |
| 6 | .465 | 4.228 | 92.288 |  |  |  |  |  |  |
| 7 | .382 | 3.473 | 95.761 |  |  |  |  |  |  |
| 8 | .363 | 3.302 | 99.064 |  |  |  |  |  |  |
| 9 | .065 | .587 | 99.651 |  |  |  |  |  |  |
| 10 | .028 | .258 | 99.909 |  |  |  |  |  |  |
| 11 | .010 | .091 | 100.000 |  |  |  |  |  |  |
| Extraction Method: Principal Component Analysis. | | | | | | | | | |

**Table S10** Principal component analysis (PCA) with varimax rotation for all heavy metal found in studied water sample.

| **Component Matrix^a^** | | | | |
| --- | --- | --- | --- | --- |
| **Elements** | **Components** | | | |
|  | **PC 1** | **PC 2** | **PC 3** | **PC 4** |
| Lead | -.212 | **.787** | -.083 | .243 |
| Cadmium | -.303 | **.512** | **.507** | -.395 |
| Chromium | **.638** | .349 | .076 | **.391** |
| Nickel | **.781** | .029 | .162 | **.483** |
| Iron | .318 | -.766 | .263 | **.339** |
| Manganese | .423 | -.062 | -.692 | -.075 |
| Zinc | **.829** | .445 | .098 | -.049 |
| Copper | **.886** | -.052 | .348 | -.140 |
| Arsenic | .321 | **.789** | -.333 | -.064 |
| Selenium | **.606** | -.089 | .233 | -.711 |
| Mercury | -.484 | .364 | .**525** | **.322** |
| Extraction Method: Principal Component Analysis  Rotation Method: Varimax with Kaiser Normalization  ^a^ Rotation converged in 8 iterations. | | | | |

**Table S11:** Single contamination factor and contamination degree of heavy metals at sampling location of Turag river.

| **Id** | **Pb** | **Cd** | **Cr** | **Ni** | **Cu** | **Zn** | **Mn** | **As** | **Hg** | **Se** | **Fe** | **C_d_** |
| --- | --- | --- | --- | --- | --- | --- | --- | --- | --- | --- | --- | --- |
| S1 | 0.043 | 0.014 | 0.003 | 0.006 | 0.039 | 0.014 | 0.033 | 0.015 | 5.731 | 0.013465 | 0.163853 | 6.07 |
| S2 | 0.049 | 0.009 | 0.002 | 0.006 | 0.024 | 0.015 | 0.211 | 0.012 | 6.594 | 0.006729 | 0.369305 | 7.30 |
| S3 | 0.032 | 0.011 | 0.001 | 0.008 | 0.049 | 0.017 | 0.302 | 0.022 | 5.050 | 0.041061 | 0.147098 | 5.68 |
| S4 | 0.001 | 0.004 | 0.003 | 0.008 | 0.072 | 0.009 | 0.074 | 0.004 | 6.197 | 0.031651 | 0.936304 | 7.34 |
| S5 | 0.042 | 0.012 | 0.034 | 0.010 | 0.091 | 0.024 | 0.217 | 0.019 | 6.312 | 0.030735 | 0.274886 | 7.07 |
| S6 | 0.051 | 0.020 | 0.004 | 0.004 | 0.032 | 0.009 | 0.026 | 0.013 | 22.725 | 0.015322 | 0.081763 | 22.98 |
| S7 | 0.027 | 0.004 | 0.014 | 0.008 | 0.078 | 0.013 | 0.164 | 0.015 | 6.893 | 0.012527 | 0.204151 | 7.43 |
| S8 | 0.019 | 0.020 | 0.004 | 0.005 | 0.095 | 0.017 | 0.213 | 0.012 | 2.252 | 0.094783 | 0.182298 | 2.91 |
| S9 | 0.012 | 0.007 | 0.006 | 0.004 | 0.013 | 0.005 | 0.031 | 0.013 | 3.223 | 0.009039 | 0.104469 | 3.43 |
| S10 | 0.032 | 0.004 | 0.004 | 0.005 | 0.017 | 0.006 | 0.551 | 0.012 | 1.933 | 0.009052 | 0.306206 | 2.88 |
| S11 | 0.030 | 0.015 | 0.006 | 0.003 | 0.014 | 0.009 | 0.027 | 0.012 | 1.358 | 0.009627 | 0.107318 | 1.59 |
| S12 | 0.048 | 0.012 | 0.004 | 0.006 | 0.022 | 0.009 | 0.061 | 0.012 | 13.680 | 0.008471 | 0.202079 | 14.07 |
| S13 | 0.027 | 0.010 | 0.005 | 0.006 | 0.018 | 0.006 | 0.057 | 0.013 | 18.379 | 0.006141 | 0.175567 | 18.70 |
| S14 | 0.038 | 0.037 | 0.003 | 0.006 | 0.017 | 0.008 | 0.057 | 0.013 | 14.870 | 0.007189 | 0.170787 | 15.23 |
| **mean** | **0.032** | **0.0127** | **0.007** | **0.006** | **0.041** | **0.011** | **0.145** | **0.0134** | **8.228** | **0.021** | **0.245** | **8.763±6.48** |
